# Hepatitis C virus transmission among people who inject drugs in the Middle East and North Africa: mathematical modeling analyses of incidence and intervention impact

**DOI:** 10.1016/j.eclinm.2024.103040

**Published:** 2025-01-15

**Authors:** Monia Makhoul, Ghina R. Mumtaz, Houssein H. Ayoub, Muhammad S. Jamil, Joumana G. Hermez, Ahmed S. Alaama, Laith J. Abu-Raddad

**Affiliations:** a Infectious Disease Epidemiology Group, Weill Cornell Medicine-Qatar, Cornell University, Doha, 24144, Qatar; b World Health Organization Collaborating Centre for Disease Epidemiology Analytics on HIV/AIDS, Sexually Transmitted Infections, and Viral Hepatitis, Weill Cornell Medicine-Qatar, Cornell University, Qatar-Foundation-Education City, Doha, 24144, Qatar; c Department of Epidemiology and Population Health, Faculty of Health Sciences, American University of Beirut, Beirut, Lebanon; d Center for Infectious Diseases Research, Faculty of Medicine, American University of Beirut, Beirut, Lebanon; e Mathematics Program, Department of Mathematics and Statistics, College of Arts and Sciences, Qatar University, Doha, Qatar; f Department of Communicable Diseases, HIV/Hepatitis/STIs Unit, World Health Organization Regional Office for the Eastern Mediterranean, Cairo, Egypt; g Department of Population Health Sciences, Weill Cornell Medicine, Cornell University, New York City, New York, 10021, USA; h Department of Public Health, College of Health Sciences, QU Health, Qatar University, Doha, Qatar; i College of Health and Life Sciences, Hamad Bin Khalifa University, Doha, Qatar

**Keywords:** HCV, Drug injection, Mathematical model, Treatment as prevention, Epidemiology

## Abstract

**Background:**

The Middle East and North Africa (MENA) region is the most affected by hepatitis C virus (HCV) infection globally. This study aimed to estimate HCV incidence among people who inject drugs (PWID) in MENA and evaluate the impact of interventions.

**Methods:**

A mathematical model was extended and applied to 13 countries with at least one data point on the population size of PWID and HCV antibody prevalence among PWID, generating estimates for the period 2024–2030. The model was calibrated using multiple datasets, primarily derived from systematic reviews and meta-analyses. Multivariable uncertainty analyses were conducted.

**Findings:**

Incidence rate among PWID in the 13 countries combined was 10.4 per 100 person-years (95% UI: 8.0–14.1), with an estimated 42,364 new infections annually (95% UI: 27,990–57,540), accounting for 16.9% (95% UI: 8.3–28.2) of all cases in these countries. These figures varied widely across countries. A 75% reduction in needle/syringe sharing decreased viremic chronic infection prevalence by 14.2% (95% UI: 11.3–17.1), incidence rate by 33.8% (95% UI: 30.2–40.5), and annual new infections by 24.4% (95% UI: 17.7–30.1). A 10% reduction in PWID numbers and a 20% reduction in injection frequency decreased chronic infection prevalence by 1.7% (95% UI: 1.4–2.5), incidence rate by 4.2% (95% UI: 3.9–4.4), and annual new infections by 11.1% (95% UI: 10.9–11.9). Achieving 75% direct-acting antiviral treatment coverage by 2030 decreased chronic infection prevalence by 65.3% (95% UI: 64.8–65.8), incidence rate by 34.5% (95% UI: 29.6–40.3), and annual new infections by 25.3% (95% UI: 19.9–29.3). Combinations of interventions reduced these epidemiologic outcomes by up to 80%.

**Interpretation:**

MENA experiences considerable HCV incidence among PWID. While the interventions showed potential, only large-scale or multi-intervention strategies can achieve meaningful reductions in HCV transmission.

**Funding:**

This publication was made possible by NPRP grant number 12S-0216-190,094 from the 10.13039/100008982Qatar National Research Fund (a member of Qatar Foundation). The authors alone are responsible for the views expressed in this publication and they do not necessarily represent the views, decisions, or policies of World Health Organization.

**Research in context**

**Evidence before this study**

The Middle East and North Africa (MENA) region has the highest global burden of hepatitis C virus (HCV) infection, a blood-borne infection that causes acute hepatitis, fibrosis, cirrhosis, and liver cancer, but is now curable with highly effective direct-acting antivirals. HCV disproportionately affects people who inject drugs (PWID). The World Health Organization has set an ambitious target to eliminate this infection by 2030. Understanding HCV incidence among PWID in MENA is essential for guiding the expansion of harm reduction interventions and developing cost-effective public health programs to eliminate this infection. A search of PubMed, from database inception to August 6, 2024, using the terms “hepatitis C virus,” “people who inject drugs,” and “Middle East and North Africa,” found no mathematical modeling studies that provide a detailed description of HCV transmission dynamics among PWID in MENA and assess the potential impact of interventions.

**Added value of this study**

This study employed mathematical modeling to estimate HCV outcomes among PWID across 13 MENA countries. The results indicated that over 42,000 new HCV infections occur annually among PWID in these countries. The incidence rate was estimated at approximately 10 per 100 person-years, indicating that 10% of PWID are newly infected each year. However, large variations were observed across countries. Modeling of various intervention scenarios demonstrated the potential to reduce viremic chronic infection prevalence, incidence rate, and the annual number of new infections by up to 70% through increased needle and syringe program coverage, and by up to 85% through expanded direct-acting antiviral treatment. Combinations of interventions yielded optimal results, even with intermediate coverage of individual strategies.

**Implications of all the available evidence**

The MENA region grapples with a high burden of HCV infection among PWID. Although interventions can have an impact, the scale of the impact is often substantially lower than the scale of the intervention due to the high-risk injecting environment and rapid turnover within this population. Achieving meaningful progress requires large-scale, multi-strategy interventions, regardless of varying HCV epidemic patterns, current resource allocation, and progress of national HCV programs in the region. Expanding harm reduction programs and increasing resource allocation are essential to meet the World Health Organization's goal of HCV elimination by 2030. However, the current response remains inadequate, constrained by a lack of political commitment and insufficient resources.

## Introduction

The Middle East and North Africa (MENA) region bears the highest burden of hepatitis C virus (HCV) infection,^1^, ^2^, ^3^ with an estimated 11.7 million people living with the infection in 2022, representing nearly a quarter of all people living with hepatitis C globally.^3^ Viremic chronic HCV infection can lead to severe complications, including acute hepatitis, fibrosis, cirrhosis, and liver cancer, straining healthcare systems.^4^, ^5^, ^6^ The development of highly effective and curative direct-acting antivirals (DAAs) offers a transformative opportunity to control HCV transmission and mitigate its disease burden.^7^, ^8^, ^9^ The increasing accessibility and affordability of DAAs, even in resource-limited settings, align with the World Health Organization's (WHO's) ambitious global targets for diagnosing, treating, and curing viral hepatitis, paving the way for HCV elimination.^3^^,^^10^, ^11^, ^12^, ^13^

HCV, a blood-borne pathogen, is predominantly transmitted through parenteral routes.^6^ Needle and syringe sharing among people who inject drugs (PWID) constitutes a major risk factor for HCV infection,^14^ with an estimated global HCV antibody (Ab) prevalence of about 50% among this population.^15^^,^^16^ MENA, situated along major drug production and trafficking routes, exhibits a vulnerability to injecting drug use.^16^, ^17^, ^18^ Targeted HCV screening programs for PWID are essential for optimizing viral hepatitis elimination efforts by maximizing the efficiency of case identification.^19^ Characterizing HCV incidence among PWID in MENA, along with its proportional contribution to overall HCV incidence, is critical for informing the expansion of harm reduction interventions and developing cost-effective, population-specific programs for HCV screening and treatment.^14^

This study utilized mathematical modeling to achieve three objectives related to HCV transmission among PWID in MENA countries. The first objective is to estimate the incidence of HCV attributable to non-sterile drug injections among PWID. The second objective is to quantify the contribution of PWID-related HCV incidence to the overall HCV incidence in the total population of each country. The third objective is to evaluate the potential impact of targeted interventions aimed at controlling transmission among PWID. This work was conducted as part of the MENA HCV Epidemiology Synthesis Project,^2^ an initiative designed to characterize the regional HCV epidemiology and inform public health priorities for policy, programming, and research.

## Methods

Thirteen MENA countries—Afghanistan, Egypt, Iran, Kuwait, Lebanon, Libya, Morocco, Oman, Pakistan, Palestine, Saudi Arabia, Syria, and Tunisia—were included in this study. Selection was based on the availability of sufficient data, defined as having at least one datapoint for each of the population size of PWID and Ab HCV prevalence among PWID in each country, to enable model calibration and application. Countries lacking these datapoints were excluded from the study. The study provided estimates for the year 2024 and projections extending through 2030.

### Mathematical model

This study builds upon an established mathematical model for infection parenteral transmission developed by Kwon et al.^20^ In our previous work,^21^ the model was adapted and extended to estimate HIV incidence among PWID in MENA. In the current study, this foundation was leveraged for a further adaptation and extension, tailoring the model to estimate HCV incidence among PWID in the same region. A description of the model parameters can be found in Table S1 of the Supplementary Material, while the model derivation is provided in Section S1.

The model assumes that needle and syringe sharing occurs within defined sharing groups, where PWID randomly share injection equipment, with each individual injecting only once per sharing event. HCV transmission is only possible in groups containing at least one chronically infected individual. The model derivation begins by defining the total population of PWID and their average injection frequency per year, and then estimating the likelihood of sharing events based on the proportion of PWID who share injections and the proportion of injections involving shared needles/syringes. Group size is incorporated as a critical factor in sharing dynamics.

For each sharing event, the model uses binomial theory to estimate the probability that a sharing group contains chronically infected, recovered, and susceptible individuals, accounting for variations in group composition. Probabilistic calculations are then applied to estimate the average number of susceptible or recovered individuals likely to inject before or after a chronically infected individual has used the injection. By aggregating these probabilities, the model calculates the expected number of HCV transmissions per sharing event. The distribution of the size of sharing groups was modeled using a gamma distribution (Section S2), with the variance set equal to its mean. This modeling choice was informed by risk behavior data.^16^^,^^17^^,^^21^, ^22^, ^23^

The model estimates the number of HCV transmissions per sharing event by incorporating data on factors including HCV Ab prevalence, HCV viremic rate, probability of HCV transmission through a contaminated needle/syringe, effectiveness of prior HCV infection in reducing the risk of reinfection, proportion of shared injections that are cleaned, effectiveness of needle/syringe cleaning in preventing HCV transmission, and number of times a needle/syringe is used before disposal. The viremic rate represents the proportion of individuals who have ever been infected with HCV and are chronically infected (Ab positive and RNA positive) out of all ever-infected individuals (Ab positive regardless of RNA status).^24^ Given detectable levels of the virus in their blood, these individuals are infectious.

Using the estimated number of transmissions per sharing event, the model subsequently estimates HCV incidence among the PWID population. This is achieved by incorporating data on the size of this population, frequency of injecting, proportion of PWID who share injections, and proportion of injections that are shared. Accordingly, the model allows for a detailed estimation of HCV transmission dynamics among PWID.

The model was applied to the total PWID population in each country. The model was also expanded to incorporate the effects of targeted interventions aimed at reducing HCV transmission. The model was developed, simulated, and analyzed using the core MATLAB R2019a platform, with the Parallel Computing Toolbox utilized to parallelize computations.^25^ High-performance computing resources from Cornell University's Red Cloud infrastructure were utilized to meet computational demands.

### Data sources and model calibration

Model parameters and their assigned values are described in Table S1. Primary sources for the country-specific parameters such as number of PWID, Ab prevalence, viremic rate, injection frequency, proportion of PWID who share injections, and proportion of injections shared included systematic reviews on HCV and HIV epidemiology and injecting behaviors among PWID in MENA.^16^, ^17^, ^18^^,^^21^^,^^22^^,^^26^^,^^27^ These reviews were predominantly conducted through the MENA HCV Epidemiology Synthesis Project and the MENA HIV Epidemiology Synthesis Project.^16^, ^17^, ^18^^,^^21^^,^^22^^,^^26^^,^^27^

Country-specific Ab prevalence was calculated by pooling all available Ab prevalence measures among PWID for each country using the DerSimonian-Laird random-effects model,^28^ applying a published methodology.^16^ Country-specific prevalence of chronic infection was calculated by multiplying Ab prevalence by the viremic rate, the latter of which was derived from a pooled estimate based on a systematic review and meta-analysis of viremic rates in MENA.^29^ HCV incidence data for the total population in each country were obtained from published modeling estimates for the year 2024.^11^^,^^30^, ^31^, ^32^

Other parameters, such as probability of HCV transmission through a contaminated needle/syringe, effectiveness of prior HCV infection in reducing reinfection risk, and effectiveness of needle/syringe cleaning in preventing transmission, were obtained from the global epidemiological literature (Table S1).^20^^,^^21^^,^^33^, ^34^, ^35^, ^36^, ^37^, ^38^, ^39^, ^40^, ^41^

### Model simulations

The model was run for each country for a “burn-in” period of 50 years, with a one-week time step. This 50-year period allowed the model to reach an endemic equilibrium for HCV transmission. Informed by data suggesting an average injection duration of about 10 years,^16^, ^17^, ^18^^,^^21^^,^^22^^,^^27^ the model assumed a yearly exit rate of 10% from the PWID population. All individuals leaving the population were assumed Ab positive, reflecting the high likelihood of HCV infection after a decade of injecting. These individuals were replaced by HCV-naïve individuals to maintain a stable population size.

For each country, the model-generated Ab prevalence at equilibrium was fitted to the empirically measured Ab prevalence. This fitting determined the shape parameter of the gamma distribution used to model sharing group size (Section S2). A nonlinear, least-square fitting method, utilizing the Nelder-Mead simplex algorithm,^42^ was employed. As expected, the fitting process typically yielded a left-skewed distribution (Fig. S1). This aligns with behavioral patterns reported in prior studies,^17^^,^^21^^,^^22^ indicating that most PWID share injections in smaller groups, with a smaller proportion engaging in sharing at larger settings like shooting galleries.

Following model fitting, estimates were generated for a range of outcomes for each country. These outcomes included the annual number of new HCV infections (incidence), incidence rate, Ab prevalence, chronic infection prevalence, and the contribution of incidence among PWID to the incidence in the total population. The latter was calculated by dividing the estimated annual number of new infections among PWID by the estimated annual number of new infections in the total population of each country.^11^^,^^30^, ^31^, ^32^ These estimated outcomes were generated for scenarios with and without interventions.

### Impact of interventions

The model was expanded to investigate the impact of interventions, assuming they are introduced on January 1, 2025, with outcomes evaluated on December 31, 2030. These interventions included: 1) needle and syringe programs (NSPs) or other behavioral interventions reducing the proportion of injections that are shared by 25%, 50%, 75%, and 95%; 2) the introduction of opioid substitution therapy (OST) (or opiate agonist therapy or other behavioral interventions), resulting in a 10% reduction in the number of PWID after one year and a 10%, 20%, or 30% reduction in injection frequency; and 3) the introduction of DAA treatment for chronically infected individuals (Section S3), scaled up at a fixed rate to reach coverage levels of 25%, 50%, 75%, and 95% by 2030. These interventions were modeled based on their effects, such as reductions in sharing or injection frequency, as these are the mechanisms that directly affect infection transmission.

DAA treatment coverage was defined as the proportion of chronically infected individuals treated by 2030 relative to the number of chronically infected individuals on January 1, 2025, when the treatment was introduced, consistent with an existing definition.^11^ Effectiveness of the treatment in real-world conditions was assumed 90%.^11^ Successfully treated individuals were assumed susceptible to reinfection but at a reduced risk compared to infection-naïve persons.^24^^,^^37^

The impact of two combination intervention packages was also investigated, designed to represent the range of potential interventions within the MENA context. The less optimistic package involved a 25% reduction in needle/syringe sharing, a 10% reduction in the number of PWID combined with a 10% decrease in injection frequency, and 25% DAA treatment coverage by 2030. The more optimistic package involved a 75% reduction in needle/syringe sharing, a 10% reduction in the number of PWID combined with a 20% decrease in injection frequency, and 95% DAA treatment coverage by 2030.

### Uncertainty analyses

To estimate the range of uncertainty surrounding the model's estimates, multivariable uncertainty analyses were conducted using Monte Carlo simulations. A total of 1000 model runs were performed for each country. These simulations drew parameter values from the uncertainty intervals defined for each model parameter, which are listed in Table S2. This number of runs was informed by previous modeling applications^30^^,^^31^^,^^43^^,^^44^ and validated by observing that, at this number of runs, the mean and distribution of outcome measures varied minimally with additional simulations.

The approach to uncertainty for model parameters varied depending on available data. Parameters with established 95% confidence intervals (CIs) had their uncertainty intervals set equal to the 95% CI. For parameters lacking a 95% CI, a 30% uncertainty interval around the point estimate was assumed. This assumption reflects a reasonable level of uncertainty based on existing modeling literature.^24^^,^^43^^,^^45^^,^^46^

During Monte Carlo sampling, a uniform probability distribution was assumed for the uncertainty intervals. The generated outcome estimates from these simulations were then used to calculate the 95% uncertainty interval (95% UI) for each outcome. Both the point estimates and their corresponding 95% UIs were reported.

### Sensitivity analysis for model validation

The model input for country-specific Ab prevalence was generated by pooling all available Ab prevalence measures in each country. This approach maximized data utility for model calibration, especially since most countries lacked sufficient time-series data for time-trend forecasting.

To validate this approach, a sensitivity analysis using an alternative method was conducted for Pakistan—a country with multiple Ab prevalence datapoints spanning two decades and the highest estimated HCV incidence among PWID in MENA. In this method, Ab prevalence data from Pakistan were divided based on the median year of data collection. Datapoints before the median year served as the training dataset for model calibration, while datapoints after the median year were used as the validation dataset to evaluate the model's predictive accuracy. Key outcomes from this sensitivity analysis, including incidence rate, annual new infections, and the contribution of incidence among PWID to total population incidence, were compared with estimates from the main analysis.

### Ethics

This study was conducted in accordance with established ethical guidelines and principles. As the data utilized were derived from publicly available sources and previously published systematic reviews, no new data collection involving human participants was undertaken. Consequently, formal ethics approval was not required for this research.

### Role of the funding source

The funders of the study had no role in study design, data collection, data analysis, data interpretation, or writing of the report. MM and LJA had full access to all the data in the study and had the final responsibility for the decision to submit for publication.

## Results

### HCV incidence among PWID

Table 1 and Fig. S2 summarize the estimated measures in the 13 countries for 2024. HCV incidence rates exhibited large variation, ranging from 3.0 (95% UI: 1.5–14.0) per 100 person-years in Syria to a high of 84.2 (95% UI: 45.5–184.5) per 100 person-years in Libya. Similarly, the annual number of new infections varied widely, with Kuwait having the lowest at 70 (95% UI: 61–274) and Pakistan the highest at 24,204 (95% UI: 11,858–38,453).

**Table 1:** Model estimates of HCV incidence among PWID in the Middle East and North Africa compared to the total population in 2024.

Country	Epidemiological measures extracted or analyzed based on the literature				Model estimates		
	Number of PWID	HCV Ab prevalence among PWID	HCV chronic infection prevalence among PWID	Number of new HCV infections among the total population per year	HCV incidence rate among PWID	Number of new HCV infections among PWID per year	Contribution of HCV incidence among PWID to the total incidence in the population
	n	% (95% CI)	% (95% CI)	n (95% UI)	per 100 person-year (95% UI)	n (95% UI)	% (95% UI)
Afghanistan	57,207 (36,767–62,490)	30.3 (23.4–37.8)	20.5 (15.8–25.6)	20,546 (11,208–38,074)	4.2 (3.0–5.7)	1704 (977–2093)	8.3 (3.1–13.1)
Egypt	93,314 (69,240–117,388)	40.5 (17.7–65.7)	27.4 (13.1–43.6)	42,588 (24,041–75,442)	6.4 (2.3–16.2)	3712 (1600–6583)	8.7 (2.6–18.3)
Iran	186,686 (104,186–269,186)	49.2 (45.1–53.4)	33.3 (30.1–36.7)	4694 (2316–13,219)	9.0 (7.6–10.6)	9033 (5240–13,258)	192.4 (49.0–372.4)
Kuwait	2300 (1850–8750)	31.2 (27.5–35.0)	21.1 (18.5–23.9)	1922 (385–7150)	4.3 (3.7–5.1)	70 (61–274)	3.7 (1.1–30.4)
Lebanon	9000 (6698–11,302)	24.6 (15.5–34.8)	16.6 (10.9–23.5)	238 (48–1532)	3.1 (1.9–5.0)	217 (124–343)	91.1 (11.1–302.5)
Libya	4446 (2948–5943)	94.2 (90.8–96.7)	63.7 (59.8–66.9)	1289 (836–2230)	84.2 (45.5–184.5)	411 (278–543)	31.9 (14.6–53.0)
Morocco	18,000 (3000–33,000)	63.3 (51.4–74.5)	42.8 (34.9–50.9)	8196 (4336–15,029)	15.5 (9.7–24.5)	1120 (225–2112)	13.7 (2.1–35.2)
Oman	4250 (2800–5700)	45.5 (34.1–57.1)	30.8 (23.4–38.5)	2564 (1317–4998)	7.8 (5.0–11.9)	190 (112–292)	7.4 (2.9–15.3)
Pakistan	430,000 (219,750–640,250)	57.3 (45.8–68.4)	38.7 (31.2–46.5)	161,306 (105,951–245,581)	12.3 (8.0–18.5)	24,204 (11,858–38,453)	15.0 (6.0–29.5)
Palestine	5000 (4350–5650)	41.6 (36.2–47.0)	28.1 (24.4–32.2)	241 (152–409)	6.7 (5.4–8.4)	204 (164–249)	84.6 (46.4–134.9)
Saudi Arabia	16,800 (11,336–22,264)	55.5 (20.5–87.6)	37.5 (15.2–57.9)	5479 (3092–10,007)	11.5 (2.8–44.7)	916 (323–1649)	16.7 (4.4–35.0)
Syria	10,000 (7750–12,250)	23.8 (0.7–62.5)	16.1 (8.9–41.4)	988 (601–1770)	3.0 (1.5–14.0)	233 (121–660)	23.6 (8.3–76.8)
Tunisia	11,000 (8462–13,750)	32.1 (22.9–42.1)	21.7 (15.8–28.5)	408 (187–1125)	4.5 (2.9–6.7)	347 (222–513)	85.1 (24.4–183.4)
All 13 countries	848,003 (479,137–1,207,923)	50.8 (44.2–57.6)	34.4 (29.9–39.2)	250,459 (154,470–416,565)	10.4 (8.0–14.1)	42,364 (27,990–57,540)	16.9 (8.3–28.2)

Ab: antibody, CI: confidence interval, HCV: hepatitis C virus, PWID: people who inject drugs, UI: uncertainty interval.

When combining the 13 countries, the overall incidence rate among PWID was 10.4 (95% UI: 8.0–14.1) per 100 person-years, with an estimated 42,364 (95% UI: 27,990–57,540) new infections annually.

The proportion of incidence among PWID compared to total incidence was 16.9% (95% UI: 8.3–28.2) across all countries combined, but it varied substantially between individual countries. In Afghanistan, Egypt, Kuwait, Morocco, Oman, Pakistan, and Saudi Arabia, incidence among PWID accounted for less than 20% of the total incidence. Conversely, in Iran, Lebanon, Palestine, and Tunisia, incidence among PWID accounted for over 80% of new cases. Notably, Iran's estimated incidence among PWID even surpassed the most recent estimate for the total population.^11^

### Impact of reducing needle/syringe sharing

**Fig. 1:**
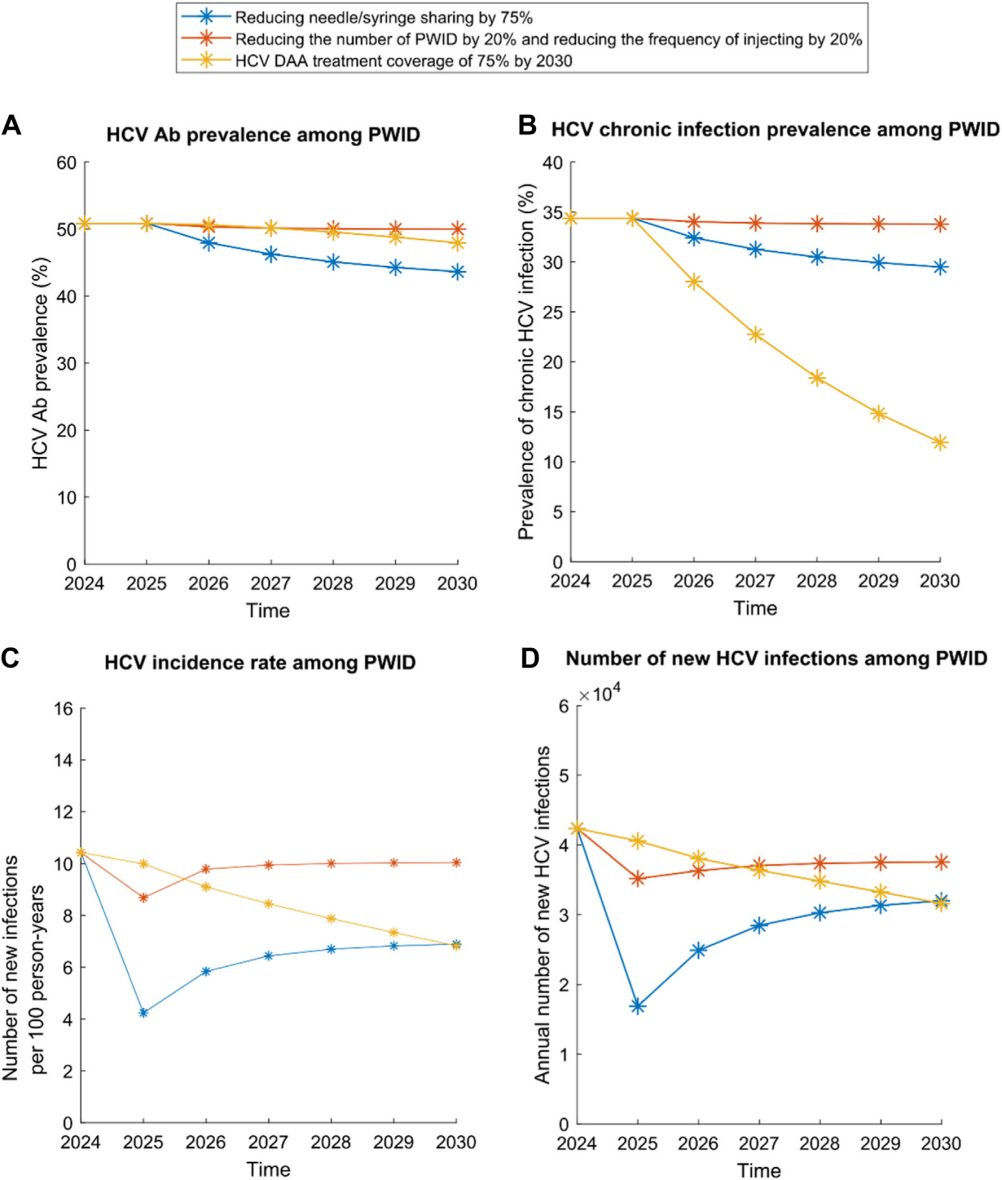
Impact of different interventions on A) HCV Ab prevalence, B) HCV chronic infection prevalence, C) HCV incidence rate, and D) number of new HCV infections among PWID in the 13 Middle East and North Africa countries combined, as interventions are introduced on January 1, 2025, with their impact assessed year by year through December 31, 2030. A version of this figure, including the 95% uncertainty intervals for the estimates, is available in Fig. S3. Ab: antibody, HCV: hepatitis C virus, PWID: people who inject drugs.

Table 2 summarizes the estimated impact of reducing needle/syringe sharing if the intervention is introduced in 2025 and assessed in 2030. Greater reductions in needle/syringe sharing resulted in a larger impact. The most substantial reductions were observed in incidence rate, followed by the annual number of new infections. Although reductions in Ab prevalence and chronic infection prevalence were considerable, they were less pronounced. The impact on incidence was greatest immediately following the intervention's introduction but decreased over time (Fig. 1 and Fig. S3).

**Table 2:** Percent reduction in HCV prevalence and incidence among PWID in the Middle East and North Africa due to reducing needle/syringe sharing.

	Afgha-nistan	Egypt	Iran	Kuwait	Lebanon	Libya	Morocco	Oman	Pakistan	Palestine	Saudi Arabia	Syria	Tunisia	All 13 countries
	% (95% UI)	% (95% UI)	% (95% UI)	% (95% UI)	% (95% UI)	% (95% UI)	% (95% UI)	% (95% UI)	% (95% UI)	% (95% UI)	% (95% UI)	% (95% UI)	% (95% UI)	% (95% UI)
**Reducing needle/syringe sharing by 25%**														
Ab prevalence	4.0 (2.3–5.8)	2.1 (0.9–4.9)	2.4 (1.4–3.8)	3.9 (2.5–5.5)	4.7 (2.6–6.6)	0.8 (0.5–1.3)	1.4 (0.8–2.7)	2.7 (1.5–4.5)	2.1 (1.1–3.6)	3.0 (1.7–4.4)	2.2 (0.8–5.6)	4.8 (1.5–7.1)	3.8 (2.0–5.7)	2.2 (1.7–3.0)
Chronic infection prevalence	4.0 (2.3–5.8)	2.1 (0.9–4.9)	2.4 (1.4–3.8)	3.9 (2.5–5.5)	4.7 (2.6–6.6)	0.8 (0.5–1.3)	1.4 (0.8–2.7)	2.7 (1.5–4.5)	2.1 (1.1–3.6)	3.0 (1.7–4.4)	2.2 (0.8–5.6)	4.8 (1.5–7.1)	3.8 (2.0–5.7)	2.2 (1.9–3.3)
Incidence rate	7.4 (3.6–12.9)	3.6 (1.8–9.4)	4.7 (2.6–8.4)	7.2 (3.7–12.2)	9.0 (4.0–15.0)	6.8 (3.0–13.9)	3.6 (2.0–6.4)	5.2 (2.8–9.7)	4.6 (2.5–8.7)	5.6 (2.9–10.0)	4.7 (2.6–11.9)	9.3 (2.9–17.6)	7.0 (3.3–12.7)	4.8 (5.0–5.9)
Number of new infections	5.9 (2.5–11.3)	2.3 (0.9–8.2)	2.7 (1.4–5.6)	5.7 (2.8–10.4)	7.7 (3.0–13.8)	0.8 (0.5–1.4)	1.5 (0.8–3.3)	3.3 (1.5–7.5)	2.3 (1.1–5.4)	3.8 (1.7–7.3)	2.4 (0.8–10.3)	8.0 (1.5–16.8)	5.5 (2.2–11.1)	2.6 (2.1–3.6)
**Reducing needle/syringe sharing by 50%**														
Ab prevalence	10.1 (6.5–13.2)	6.1 (2.6–11.9)	6.6 (4.1–9.4)	10.0 (6.9–12.6)	11.5 (7.2–14.7)	2.3 (1.4–3.5)	4.2 (2.2–7.1)	7.4 (4.4–10.8)	5.8 (3.3–9.2)	8.0 (4.9–10.8)	6.0 (2.4–12.8)	11.7 (4.3–15.5)	9.7 (5.8–13.1)	6.3 (4.6–8.2)
Chronic infection prevalence	10.1 (6.5–13.2)	6.1 (2.6–11.9)	6.6 (4.1–9.4)	10.0 (6.9–12.6)	11.5 (7.2–14.7)	2.3 (1.4–3.5)	4.2 (2.2–7.1)	7.4 (4.4–10.8)	5.8 (3.3–9.2)	8.0 (4.9–10.8)	6.0 (2.4–12.8)	11.7 (4.3–15.5)	9.7 (5.8–13.1)	6.2 (4.9–8.0)
Incidence rate	21.0 (11.2–30.7)	11.0 (5.5–25.2)	14.0 (7.7–22.4)	20.5 (11.7–29.3)	24.4 (12.6–34.3)	17.6 (8.4–31.9)	10.3 (5.7–17.6)	15.4 (8.4–24.9)	13.5 (7.4–22.7)	16.4 (8.9–25.2)	13.7 (7.5–29.1)	24.9 (8.7–38.0)	20.0 (10.2–30.3)	13.8 (13.3–18.0)
Number of new infections	17.7 (8.5–27.8)	7.7 (2.7–22.8)	9.0 (4.4–16.4)	17.2 (9.3–25.8)	21.8 (9.9–32.5)	2.4 (1.4–4.2)	4.7 (2.2–10.5)	10.7 (4.8–20.3)	7.5 (3.5–15.8)	12.1 (5.6–20.1)	7.9 (2.5–26.4)	22.4 (4.7–36.6)	16.6 (7.1–27.4)	8.4 (5.8–11.6)
**Reducing needle/syringe sharing by 75%**														
Ab prevalence	19.8 (15.1–23.0)	14.3 (7.5–22.0)	14.9 (10.7–18.5)	19.6 (15.7–22.3)	21.5 (16.2–24.7)	6.0 (3.8–8.4)	10.7 (6.3–15.3)	16.0 (11.2–20.3)	13.5 (8.9–18.2)	16.9 (12.4–20.3)	13.9 (6.7–22.8)	21.7 (10.9–25.5)	19.3 (14.0–23.0)	14.2 (10.9–17.2)
Chronic infection prevalence	19.8 (15.1–23.0)	14.3 (7.5–22.0)	14.9 (10.7–18.5)	19.6 (15.7–22.3)	21.5 (16.2–24.7)	6.0 (3.8–8.4)	10.7 (6.3–15.3)	16.0 (11.2–20.3)	13.5 (8.9–18.2)	16.9 (12.4–20.3)	13.9 (6.7–22.8)	21.7 (10.9–25.5)	19.3 (14.0–23.0)	14.2 (11.3–17.1)
Incidence rate	45.0 (31.5–54.9)	30.3 (16.5–50.2)	34.6 (22.4–44.9)	44.5 (32.4–53.3)	49.3 (33.9–59.2)	37.6 (21.4–55.6)	27.2 (16.1–38.9)	36.9 (24.7–48.2)	33.3 (21.3–45.2)	38.6 (25.7–48.2)	33.7 (21.0–53.6)	49.9 (24.6–62.7)	43.8 (28.9–54.5)	33.8 (30.2–40.5)
Number of new infections	40.7 (26.2–51.4)	24.2 (9.0–47.7)	26.1 (15.3–36.8)	39.9 (28.0–49.0)	46.0 (29.2–57.2)	7.2 (4.0–11.6)	15.6 (7.2–27.7)	29.3 (16.5–42.3)	22.6 (11.7–35.9)	31.9 (19.0–42.2)	23.5 (8.1–50.6)	46.8 (15.9–61.3)	39.1 (22.9–51.2)	24.4 (17.7–30.1)
**Reducing needle/syringe sharing by 95%**														
Ab prevalence	33.3 (30.7–34.8)	30.0 (23.3–34.5)	30.0 (26.7–32.2)	33.2 (31.0–34.4)	34.2 (31.4–35.6)	18.4 (14.2–22.0)	26.2 (21.0–30.1)	30.8 (27.1–33.4)	28.8 (24.4–32.0)	31.5 (28.4–33.3)	29.1 (20.9–34.7)	34.3 (26.6–35.9)	33.0 (29.8–34.8)	29.3 (26.1–31.3)
Chronic infection prevalence	33.3 (30.7–34.8)	30.0 (23.3–34.5)	30.0 (26.7–32.2)	33.2 (31.0–34.4)	34.2 (31.4–35.6)	18.4 (14.2–22.0)	26.2 (21.0–30.1)	30.8 (27.1–33.4)	28.8 (24.4–32.0)	31.5 (28.4–33.3)	29.1 (20.9–34.7)	34.3 (26.6–35.9)	33.0 (29.8–34.8)	29.2 (26.5–31.2)
Incidence rate	80.7 (72.7–85.5)	71.3 (57.1–84.4)	72.9 (63.6–79.0)	80.3 (73.5–84.5)	83.3 (74.4–88.1)	71.5 (56.3–82.8)	66.3 (55.0–74.7)	74.7 (65.8–81.4)	71.2 (61.0–78.6)	76.1 (67.5–81.5)	71.6 (59.7–85.1)	83.7 (65.0–89.7)	79.9 (70.8–85.3)	71.8 (66.9–76.9)
Number of new infections	78.1 (68.6–83.9)	65.9 (44.7–82.9)	65.8 (55.4–73.4)	77.6 (69.8–82.3)	81.6 (71.2–87.3)	29.2 (20.4–38.3)	53.2 (38.2–65.7)	68.8 (56.1–78.3)	61.4 (47.9–72.8)	71.2 (60.7–77.8)	62.5 (37.1–83.6)	82.1 (54.5–89.2)	77.0 (65.6–83.8)	63.4 (54.3–69.3)

The intervention is introduced on January 1, 2025, with its impact assessed on December 31, 2030.

Ab: antibody, HCV: hepatitis C virus, PWID: people who inject drugs, UI: uncertainty interval.

A 75% decrease in needle/syringe sharing across all countries reduced Ab prevalence by 14.2% (95% UI: 10.9–17.2), chronic infection prevalence by 14.2% (95% UI: 11.3–17.1), incidence rate by 33.8% (95% UI: 30.2–40.5), and annual number of new infections by 24.4% (95% UI: 17.7–30.1).

### Impact of opioid substitution therapy

Table 3 summarizes the estimated impact of introducing OST if the intervention is implemented in 2025 and assessed in 2030. Greater reductions in the injection frequency resulted in a larger impact. The largest reductions were observed in the annual number of new infections, while reductions in incidence rate, Ab prevalence, and chronic infection prevalence were substantially smaller. The impact on incidence was greatest immediately following the intervention's introduction but decreased over time (Fig. 1).

**Table 3:** Percent reduction in HCV prevalence and incidence among PWID in the Middle East and North Africa due to opioid substitution therapy (OST) resulting in a 10% reduction in the number of PWID and a 10%, 20%, or 30% reduction in injection frequency.

	Afghanistan	Egypt	Iran	Kuwait	Lebanon	Libya	Morocco	Oman	Pakistan	Palestine	Saudi Arabia	Syria	Tunisia	All 13 countries
	% (95% UI)	% (95% UI)	% (95% UI)	% (95% UI)	% (95% UI)	% (95% UI)	% (95% UI)	% (95% UI)	% (95% UI)	% (95% UI)	% (95% UI)	% (95% UI)	% (95% UI)	% (95% UI)
**Reducing the number of PWID by 10% and reducing the frequency of injecting by 10%**														
Ab prevalence	1.4 (0.8–2.1)	0.7 (0.3–1.7)	0.8 (0.5–1.3)	1.4 (0.8–2.0)	1.7 (0.9–2.4)	0.3 (0.2–0.4)	0.5 (0.3–0.9)	0.9 (0.5–1.6)	0.7 (0.4–1.3)	1.0 (0.6–1.6)	0.7 (0.3–2.0)	1.7 (0.5–2.6)	1.3 (0.7–2.1)	0.8 (0.6–1.1)
Chronic infection prevalence	1.4 (0.8–2.1)	0.7 (0.3–1.7)	0.8 (0.5–1.3)	1.4 (0.8–2.0)	1.7 (0.9–2.4)	0.3 (0.2–0.4)	0.5 (0.3–0.9)	0.9 (0.5–1.6)	0.7 (0.4–1.3)	1.0 (0.6–1.6)	0.7 (0.3–2.0)	1.7 (0.5–2.6)	1.3 (0.7–2.1)	0.8 (0.6–1.1)
Incidence rate	2.5 (1.2–4.7)	1.2 (0.6–3.2)	1.6 (0.9–2.9)	2.4 (1.2–4.4)	3.1 (1.3–5.5)	2.4 (1.0–5.2)	1.2 (0.7–2.2)	1.7 (0.9–3.4)	1.6 (0.9–3.0)	1.9 (1.0–3.5)	1.6 (0.9–4.3)	3.2 (1.0–6.7)	2.3 (1.1–4.6)	1.6 (1.9–2.1)
Number of new infections	11.3 (10.3–13.2)	10.2 (9.8–12.0)	10.3 (10.0–11.2)	11.2 (10.3–12.8)	11.9 (10.4–14.1)	9.8 (9.7–9.9)	10.0 (9.8–10.5)	10.5 (10.0–11.8)	10.2 (9.9–11.1)	10.6 (10.0–11.8)	10.2 (9.8–12.8)	12.0 (10.0–15.3)	12.0 (10.2–13.1)	10.2 (10.1–10.6)
**Reducing the number of PWID by 10% and reducing the frequency of injecting by 20%**														
Ab prevalence	3.1 (1.7–4.5)	1.6 (0.7–3.8)	1.8 (1.1–2.9)	3.0 (1.9–4.2)	3.6 (2.0–5.1)	0.6 (0.3–1.0)	1.1 (0.6–2.0)	2.1 (1.1–3.4)	1.6 (0.8–2.8)	2.3 (1.3–3.4)	1.6 (0.6–4.3)	3.7 (1.1–5.5)	2.9 (1.5–4.4)	1.7 (1.3–2.3)
Chronic infection prevalence	3.1 (1.7–4.5)	1.6 (0.7–3.8)	1.8 (1.1–2.9)	3.0 (1.9–4.2)	3.6 (2.0–5.1)	0.6 (0.3–1.0)	1.1 (0.6–2.0)	2.1 (1.1–3.4)	1.6 (0.8–2.8)	2.3 (1.3–3.4)	1.6 (0.6–4.3)	3.7 (1.1–5.5)	2.9 (1.5–4.4)	1.7 (1.4–2.5)
Incidence rate	5.6 (2.7–10.0)	2.7 (1.4–7.2)	3.6 (2.0–6.4)	5.5 (2.8–9.4)	6.9 (3.0–11.7)	5.2 (2.3–10.8)	2.7 (1.5–4.8)	3.9 (2.1–7.4)	3.5 (1.9–6.6)	4.2 (2.2–7.6)	3.5 (2.0–9.2)	7.1 (2.2–13.9)	5.3 (2.5–9.8)	4.2 (3.9–4.4)
Number of new infections	13.5 (11.2–17.4)	11.1 (10.1–15.2)	11.4 (10.5–13.3)	13.4 (11.4–16.7)	14.8 (11.5–19.2)	10.1 (9.8–10.5)	10.5 (10.0–11.7)	11.7 (10.6–14.6)	11.1 (10.3–13.2)	12.1 (10.7–14.6)	11.1 (10.1–16.7)	15.0 (10.5–21.5)	13.2 (11.0–17.3)	11.1 (10.9–11.9)
**Reducing the number of PWID by 10% and reducing the frequency of injecting by 30%**														
Ab prevalence	5.0 (3.0–7.1)	2.7 (1.1–6.1)	3.0 (1.8–4.7)	4.9 (3.2–6.7)	5.9 (3.3–8.0)	1.0 (0.6–1.6)	1.9 (1.0–3.4)	3.5 (1.9–5.5)	2.7 (1.4–4.6)	3.8 (2.2–5.5)	2.8 (1.1–6.8)	6.0 (1.9–8.6)	4.8 (2.6–7.0)	3.0 (2.1–3.7)
Chronic infection prevalence	5.0 (3.0–7.1)	2.7 (1.1–6.1)	3.0 (1.8–4.7)	4.9 (3.2–6.7)	5.9 (3.3–8.0)	1.0 (0.6–1.6)	1.9 (1.0–3.4)	3.5 (1.9–5.5)	2.7 (1.4–4.6)	3.8 (2.2–5.5)	2.8 (1.1–6.8)	6.0 (1.9–8.6)	4.8 (2.6–7.0)	3.0 (2.4–4.0)
Incidence rate	9.6 (4.7–16.0)	4.6 (2.4–12.0)	6.1 (3.3–10.7)	9.3 (4.9–15.2)	11.6 (5.2–18.5)	8.5 (3.8–17.1)	4.5 (2.5–8.1)	6.8 (3.5–12.2)	5.9 (3.2–11.0)	7.2 (3.8–12.5)	6.0 (3.4–14.9)	11.9 (3.8–21.5)	9.0 (4.3–15.9)	5.8 (6.3–7.8)
Number of new infections	16.5 (12.5–22.3)	12.3 (10.6–19.1)	12.8 (11.2–16.1)	16.3 (12.8–21.3)	18.6 (13.1–25.1)	10.5 (10.1–11.1)	11.3 (10.4–13.4)	13.4 (11.3–18.2)	12.2 (10.8–15.8)	14.0 (11.6–18.0)	12.4 (10.5–21.3)	18.9 (11.3–28.1)	16.0 (12.1–22.1)	12.2 (11.9–13.8)

The intervention is introduced on January 1, 2025, with its impact assessed on December 31, 2030.

Ab: antibody, HCV: hepatitis C virus, PWID: people who inject drugs, UI: uncertainty interval.

A 10% reduction in the number of PWID and a 20% reduction in the injection frequency across all countries reduced Ab prevalence by 1.7% (95% UI: 1.3–2.3), chronic infection prevalence by 1.7% (95% UI: 1.4–2.5), incidence rate by 4.2% (95% UI: 3.9–4.4), and annual number of new infections by 11.1% (95% UI: 10.9–11.9).

### Impact of DAA treatment for chronically infected individuals

Table 4 summarizes the estimated impact of DAA treatment for chronically infected PWID if the intervention is introduced in 2025, scaled up to reach the target coverage by 2030, and assessed in 2030. Greater coverage for this intervention resulted in a larger impact. The largest reductions were observed in chronic infection prevalence, followed by incidence rate and annual number of new infections. The reductions in Ab prevalence were substantially lower. The impact increased over time as the intervention coverage expanded (Fig. 1).

**Table 4:** Percent reduction in HCV prevalence and incidence among PWID in the Middle East and North Africa due to introducing HCV treatment with direct-acting antivirals for chronically infected PWID.

	Afgha-nistan	Egypt	Iran	Kuwait	Lebanon	Libya	Morocco	Oman	Pakistan	Palestine	Saudi Arabia	Syria	Tunisia	All 13 countries
	% (95% UI)	% (95% UI)	% (95% UI)	% (95% UI)	% (95% UI)	% (95% UI)	% (95% UI)	% (95% UI)	% (95% UI)	% (95% UI)	% (95% UI)	% (95% UI)	% (95% UI)	% (95% UI)
**HCV DAA treatment coverage of 25% by 2030**														
Ab prevalence	1.8 (1.2–2.4)	1.1 (0.3–2.2)	1.1 (0.7–1.6)	1.8 (1.2–2.3)	2.1 (1.3–2.8)	0.2 (0.1–0.4)	0.7 (0.4–1.2)	1.3 (0.8–1.9)	1.0 (0.5–1.6)	1.4 (0.9–1.9)	1.0 (0.4–2.4)	2.2 (0.7–2.9)	1.7 (1.0–2.4)	1.1 (0.8–1.4)
Chronic infection prevalence	20.8 (20.3–21.3)	20.2 (19.6–21.2)	20.3 (19.9–20.7)	20.8 (20.4–21.2)	21.1 (20.4–21.6)	19.5 (19.4–19.7)	19.9 (19.6–20.3)	20.4 (20.0–20.9)	20.1 (19.8–20.6)	20.5 (20.1–20.9)	20.2 (19.7–21.3)	21.1 (19.9–21.7)	20.8 (20.2–21.3)	20.3 (20.1–20.5)
Incidence rate	9.2 (5.9–12.6)	6.1 (3.0–10.8)	7.2 (4.4–10.7)	9.0 (6.2–12.2)	10.3 (6.8–13.9)	17.9 (6.1–24.0)	6.8 (3.7–11.6)	7.6 (4.7–11.2)	7.4 (4.5–11.1)	7.8 (4.9–11.2)	7.3 (4.6–14.2)	10.5 (5.2–15.3)	8.9 (5.8–12.7)	7.8 (6.3–9.5)
Number of new infections	7.7 (4.5–11.1)	4.1 (0.9–9.6)	4.4 (2.6–6.9)	7.5 (4.8–10.4)	9.1 (5.1–13.1)	0.9 (0.4–1.5)	2.6 (1.3–4.7)	5.1 (2.8–8.3)	3.8 (2.0–6.6)	5.6 (3.2–8.3)	3.9 (1.3–10.7)	9.4 (2.7–14.7)	7.3 (3.9–11.0)	4.1 (3.2–5.0)
**HCV DAA treatment coverage of 50% by 2030**														
Ab prevalence	4.4 (3.0–5.6)	2.8 (0.6–5.3)	2.9 (1.9–3.9)	4.3 (3.1–5.4)	5.0 (3.3–6.3)	0.7 (0.3–1.1)	1.8 (1.0–3.0)	3.2 (2.0–4.6)	2.5 (1.5–3.9)	3.5 (2.3–4.6)	2.6 (1.0–5.5)	5.1 (1.9–6.6)	4.3 (2.7–5.6)	2.8 (2.0–3.4)
Chronic infection prevalence	42.8 (41.9–43.5)	41.8 (40.5–43.3)	41.9 (41.3–42.5)	42.7 (42.0–43.4)	43.2 (42.1–43.9)	40.5 (40.4–40.8)	41.2 (40.7–41.9)	42.1 (41.3–42.9)	41.6 (41.0–42.5)	42.2 (41.5–42.9)	41.7 (40.7–43.5)	43.2 (41.3–44.1)	42.7 (41.8–43.5)	41.8 (41.4–42.4)
Incidence rate	22.1 (15.2–28.2)	15.4 (7.7–25.3)	17.6 (11.8–24.0)	21.8 (16.0–27.5)	24.4 (17.1–30.7)	32.9 (15.4–40.6)	15.9 (9.7–24.0)	18.5 (12.2–25.3)	17.5 (11.8–24.5)	19.1 (13.0–25.3)	17.6 (12.0–28.2)	24.7 (13.4–33.1)	21.5 (15.0–28.3)	18.2 (15.0–21.9)
Number of new infections	19.2 (12.3–25.6)	11.4 (3.3–23.2)	12.1 (7.7–17.2)	18.8 (13.0–24.2)	22.2 (13.7–29.3)	2.9 (1.6–4.8)	7.6 (4.0–12.7)	13.6 (8.1–20.3)	10.5 (6.0–16.7)	14.8 (9.3–20.3)	10.9 (4.1–24.9)	22.6 (7.8–32.1)	18.4 (11.1–25.4)	11.4 (8.6–13.8)
**HCV DAA treatment coverage of 75% by 2030**														
Ab prevalence	8.6 (6.3–10.4)	5.9 (2.0–9.9)	6.0 (4.2–7.7)	8.5 (6.5–10.0)	9.5 (6.8–11.4)	1.7 (1.0–2.6)	4.1 (2.4–6.2)	6.6 (4.4–8.8)	5.3 (3.4–7.6)	7.1 (5.0–8.8)	5.5 (2.4–10.2)	9.7 (4.3–11.9)	8.3 (5.8–10.3)	5.7 (4.3–6.9)
Chronic infection prevalence	66.3 (65.5–67.0)	65.3 (63.9–66.8)	65.4 (64.7–66.0)	66.3 (65.6–66.8)	66.7 (65.7–67.4)	63.8 (63.5–64.1)	64.7 (64.1–65.4)	65.6 (64.8–66.4)	65.1 (64.4–66.0)	65.8 (65.0–66.4)	65.2 (64.1–66.9)	66.7 (64.8–67.6)	66.2 (65.3–67.0)	65.3 (64.8–65.8)
Incidence rate	41.5 (32.0–49.0)	31.5 (19.9–45.8)	34.3 (26.0–42.4)	41.1 (33.2–47.7)	44.7 (34.3–52.2)	47.5 (30.3–54.5)	30.4 (21.4–40.2)	35.8 (26.6–44.5)	33.7 (25.3–42.5)	37.0 (28.1–44.6)	33.9 (25.4–48.1)	45.2 (27.8–54.9)	40.7 (31.2–48.8)	34.5 (29.6–40.3)
Number of new infections	37.9 (27.6–45.8)	25.9 (12.6–43.4)	26.8 (19.0–34.3)	37.3 (28.7–44.0)	41.9 (29.9–50.5)	8.3 (5.1–12.3)	18.5 (11.0–27.4)	29.2 (19.8–38.9)	23.8 (15.4–33.7)	31.2 (22.0–38.7)	24.6 (11.1–45.2)	42.6 (19.1–53.9)	36.7 (25.4–45.7)	25.3 (19.9–29.3)
**HCV DAA treatment coverage of 95% by 2030**														
Ab prevalence	15.3 (12.3–17.3)	11.7 (6.6–16.9)	11.8 (9.1–14.0)	15.1 (12.6–16.8)	16.4 (13.0–18.5)	4.3 (2.9–6.0)	8.9 (5.8–11.9)	12.6 (9.4–15.4)	10.8 (7.7–13.8)	13.3 (10.4–15.3)	11.0 (5.8–17.1)	16.6 (9.2–19.1)	14.9 (11.6–17.3)	11.3 (9.0–13.0)
Chronic infection prevalence	87.0 (86.5–87.3)	86.4 (85.6–87.2)	86.4 (86.0–86.8)	86.9 (86.6–87.2)	87.1 (86.6–87.5)	85.3 (85.1–85.5)	86.0 (85.5–86.4)	86.6 (86.1–87.0)	86.3 (85.8–86.7)	86.7 (86.2–87.0)	86.3 (85.5–87.2)	87.2 (86.0–87.5)	86.9 (86.4–87.3)	86.4 (86.1–86.6)
Incidence rate	67.8 (59.1–73.6)	57.9 (43.1–71.7)	60.0 (51.8–66.8)	67.4 (60.4–72.5)	70.7 (61.2–76.5)	64.2 (54.1–68.2)	54.3 (44.6–63.1)	61.8 (52.6–69.3)	58.6 (50.0–66.5)	63.1 (54.4–69.0)	59.0 (50.1–73.2)	71.2 (53.4–78.7)	67.0 (57.2–73.5)	59.4 (53.7–65.0)
Number of new infections	64.7 (54.7–71.3)	52.5 (33.7–69.9)	52.7 (43.1–60.2)	64.2 (55.9–69.7)	68.5 (57.2–75.3)	23.1 (16.2–30.2)	41.8 (29.9–52.9)	55.5 (44.2–65.1)	48.9 (37.2–59.6)	57.8 (47.9–64.8)	49.9 (29.3–70.8)	69.0 (43.1–77.8)	63.6 (52.1–71.2)	50.7 (43.0–55.7)

The intervention is introduced on January 1, 2025, with its impact assessed on December 31, 2030.

Ab: antibody, DAA: direct-acting antivirals, HCV: hepatitis C virus, PWID: people who inject drugs, UI: uncertainty interval.

A 75% treatment coverage by 2030 across all countries reduced Ab prevalence by 5.7% (95% UI: 4.3–6.9), chronic infection prevalence by 65.3% (95% UI: 64.8–65.8), incidence rate by 34.5% (95% UI: 29.6–40.3), and annual number of new infections by 25.3% (95% UI: 19.9–29.3).

### Impact of combined three-intervention packages

Table 5 summarizes the estimated impact of two packages of the three interventions: the less optimistic package and the more optimistic package. In both packages, the largest reductions were observed in chronic infection prevalence, followed by incidence rate and annual number of new infections. The reductions in Ab prevalence were substantially lower. The reductions in chronic infection prevalence, incidence rate, and annual number of new infections were approximately 20% for the less optimistic package and 80% for the more optimistic package.

**Table 5:** Percent reduction in HCV prevalence and incidence among PWID in the Middle East and North Africa under modeled scenarios for three-intervention packages.

	Afgha-nistan	Egypt	Iran	Kuwait	Lebanon	Libya	Morocco	Oman	Pakistan	Palestine	Saudi Arabia	Syria	Tunisia	All 13 countries
	% (95% UI)	% (95% UI)	% (95% UI)	% (95% UI)	% (95% UI)	% (95% UI)	% (95% UI)	% (95% UI)	% (95% UI)	% (95% UI)	% (95% UI)	% (95% UI)	% (95% UI)	% (95% UI)
**Less optimistic scenario: Reducing needle/syringe sharing by 25%, reducing the number of PWID by 10%, reducing the frequency of injecting by 10%, and introducing HCV DAA treatment at a coverage of 25% by 2030**														
Ab prevalence	7.8 (5.1–10.3)	4.7 (1.9–9.2)	5.0 (3.1–7.1)	7.7 (5.3–9.8)	8.9 (5.6–11.6)	1.5 (0.9–2.3)	3.2 (1.7–5.3)	5.6 (3.3–8.3)	4.4 (2.5–6.9)	6.1 (3.8–8.3)	4.6 (1.8–10.0)	9.1 (3.3–12.2)	7.5 (4.5–10.2)	4.7 (3.4–6.2)
Chronic infection prevalence	28.6 (26.4–30.4)	26.1 (23.6–29.3)	26.4 (24.6–27.7)	28.5 (26.6–30.1)	29.4 (27.0–31.6)	23.7 (22.3–23.5)	25.0 (23.2–26.0)	26.9 (24.8–28.6)	25.9 (24.0–27.5)	27.2 (25.3–28.7)	26.0 (23.5–29.8)	29.6 (25.3–32.2)	28.3 (26.1–30.5)	26.2 (25.1–27.0)
Incidence rate	22.6 (14.2–30.9)	14.1 (7.2–26.2)	16.8 (11.2–23.9)	22.2 (15.0–29.8)	25.6 (15.8–34.2)	27.4 (18.8–31.1)	14.0 (8.9–20.5)	17.9 (11.4–26.0)	16.5 (11.1–23.7)	18.8 (12.0–26.0)	16.6 (11.7–29.3)	26.0 (12.5–37.5)	21.8 (13.6–30.7)	17.0 (14.9–21.0)
Number of new infections	27.1 (19.6–34.7)	18.8 (12.7–31.0)	19.7 (15.4–25.3)	26.6 (20.3–33.2)	30.3 (21.0–39.0)	12.0 (10.8–13.5)	15.5 (12.5–20.3)	21.1 (15.8–28.5)	18.2 (14.1–24.8)	22.4 (16.8–28.5)	18.6 (12.7–33.5)	30.8 (15.8–42.2)	26.2 (18.4–34.5)	19.0 (16.4–21.5)
**More optimistic scenario: Reducing needle/syringe sharing by 75%, reducing the number of PWID by 10%, reducing the frequency of injecting by 20%, and introducing HCV DAA treatment at a coverage of 95% by 2030**														
Ab prevalence	29.7 (26.5–31.6)	25.7 (18.6–31.2)	25.7 (22.1–28.2)	29.5 (26.9–31.1)	30.8 (27.3–32.7)	13.1 (9.6–16.5)	21.5 (16.4–25.6)	26.7 (22.5–29.7)	24.2 (19.6–27.9)	27.4 (24.0–29.6)	24.6 (16.0–31.4)	31.0 (22.0–33.1)	29.3 (25.6–31.5)	24.9 (21.4–27.2)
Chronic infection prevalence	85.0 (84.3–85.4)	84.1 (82.6–85.3)	84.1 (83.4–84.6)	84.9 (84.4–85.3)	85.2 (84.5–85.6)	81.4 (80.7–82.1)	83.2 (82.1–84.1)	84.3 (83.4–85.0)	83.8 (82.8–84.6)	84.5 (83.8–85.0)	83.9 (82.0–85.3)	85.2 (83.3–85.7)	84.9 (84.1–85.4)	83.9 (83.3–84.4)
Incidence rate	84.9 (79.0–88.4)	77.9 (67.6–87.7)	78.9 (72.7–83.2)	84.6 (79.7–87.6)	86.9 (80.5–90.4)	77.8 (72.2–82.7)	73.9 (66.5–80.0)	80.2 (73.7–85.3)	77.5 (70.8–82.9)	81.3 (75.1–85.3)	77.9 (70.4–88.2)	87.2 (73.6–91.7)	84.3 (77.5–88.4)	78.0 (74.3–81.9)
Number of new infections	84.3 (77.8–88.2)	75.9 (60.1–87.8)	75.4 (68.2–80.6)	83.9 (78.7–87.1)	86.8 (79.6–90.7)	45.0 (36.9–52.5)	66.2 (55.0–75.1)	77.6 (68.7–84.1)	72.1 (62.2–80.1)	79.3 (72.4–83.8)	72.9 (52.9–88.1)	87.1 (67.4–92.1)	83.5 (75.7–88.1)	73.6 (66.8–77.7)

The intervention package is introduced on January 1, 2025, with its impact assessed on December 31, 2030.

Ab: antibody, DAA: direct-acting antivirals, HCV: hepatitis C virus, PWID: people who inject drugs, UI: uncertainty interval.

### Sensitivity analysis for model validation

Fig. S4 presents the model predictions for Ab prevalence from the sensitivity analysis alongside the Ab prevalence observed in the validation dataset for Pakistan. The figure also compares predictions for key epidemiological outcomes from the sensitivity analysis with those from the main analysis. Across all comparisons, predictions from the sensitivity analysis aligned closely with those from the validation dataset and the main analysis, supporting the reliability of the model's outcomes.

## Discussion

Over 40,000 HCV infections occur annually among PWID in the 13 countries studied, which comprise the majority of the MENA region's population.^47^ The incidence rate was estimated at approximately 10 per 100 person-years, indicating that 10% of PWID are newly infected with HCV each year. However, this rate varied substantially between countries, reflecting differences in PWID typologies and injecting risk environments across MENA.^16^, ^17^, ^18^^,^^21^^,^^22^^,^^27^ The eastern part of the MENA region, particularly Afghanistan, Iran, and Pakistan, exhibits high incidence rates and accounts for the majority of new infections (Fig. S2).

Despite the high incidence of infection in MENA, less than 20% of the total incidence occurs among PWID, consistent with an earlier estimate.^14^ This is in contrast to high-income countries where the majority of HCV infections occur among PWID.^14^ This finding aligns with epidemiological evidence indicating that most HCV infections in most MENA countries are related to healthcare exposures.^2^^,^^19^^,^^43^^,^^48^, ^49^, ^50^, ^51^, ^52^, ^53^, ^54^, ^55^ Consequently, none of the countries are projected to meet WHO's 2030 targets solely through interventions among PWID; additional interventions among non-PWID groups are also essential to reach these targets.

The modeled interventions had a considerable impact on reducing incidence rate, annual number of new infections, and prevalence of chronic infection. However, the scale of impact was generally substantially lower than the scale of the intervention. For example, a 75% decrease in needle/syringe sharing across all countries led to only a 34% reduction in incidence rate and a 24% reduction in annual number of new infections. This disparity between the scale of intervention and impact is due to the high-risk injecting environment among PWID, where exposure to the infection remains prevalent despite interventions. While reducing injection frequency can delay when an infection is acquired, PWID are still at risk of acquiring the infection as long as some sharing persists. Even if an intervention reduces the number of infectious individuals in a sharing group to one, that individual can still infect all others within the group.

Another factor contributing to this disparity is the short injecting career and rapid turnover within the PWID population. Informed by data,^16^, ^17^, ^18^^,^^21^^,^^22^^,^^27^ 10% of PWID were assumed to stop injecting each year, being replaced by new, uninfected individuals who are susceptible to infection. Moreover, when an intervention reduces incidence, it decreases the number of infectious individuals but increases the number of susceptible individuals who can become infected. This dynamic results in the initial impact of interventions, such as reducing needle/syringe sharing and OST, being greatest at the onset (Fig. 1). However, incidence rebounds afterward due to the influx of new susceptible individuals until a new post-intervention plateau is reached.

These findings indicate that only large-scale interventions or combinations of multiple interventions can have a meaningful impact on HCV infection among PWID, confirming similar observations from modeling studies conducted in other regions.^56^, ^57^, ^58^, ^59^, ^60^ Despite evidence of expanding HIV epidemics among PWID in MENA,^17^^,^^18^^,^^61^, ^62^, ^63^ and the demonstrated benefits of investment in HCV elimination programs,^13^^,^^64^, ^65^, ^66^ the response to HCV and HIV among PWID remains limited. Policymakers remain hesitant to allocate resources specifically for PWID interventions.^18^^,^^22^^,^^67^^,^^68^ When programs and services are available, they are typically provided by community-based organizations, which often lack the funding and legal protection needed to offer comprehensive prevention and treatment interventions.^22^^,^^67^

This lack of political commitment not only hinders the development of new programs but also undermines existing ones. For instance, several harm reduction programs have been forced to reduce or cease operations due to funding losses.^69^ Currently, NSPs appear to be operational in only eight countries, and opioid agonist therapy services are available in ten.^70^ Iran appears to be the only country that has extended NSP services to prisons and established gender-sensitive harm reduction services.^17^^,^^70^ Whether the recent successes of national HCV elimination programs targeting the general population in the region, particularly in Egypt and Pakistan,^3^^,^^65^^,^^71^ can be expanded into effective programs for PWID remains to be seen.

This study and its estimates have caveats and limitations. Estimations were only possible for 13 out of the 23 MENA countries due to the lack of sufficient data needed to apply the model. However, the included countries account for approximately 75% of the total MENA population^47^ and the population of PWID in the region.^2^^,^^16^^,^^17^^,^^21^^,^^22^^,^^26^^,^^27^^,^^72^ While estimates were provided for the 13 countries combined, these estimates are influenced by countries with large populations, particularly Pakistan, with its large PWID population (Table 1). While the study provided estimates for initiating programs and allocating resources, these estimates represent an ongoing effort that requires regular updates as new input data or revisions to existing data become available. Such updates will enhance the reliability and precision of the estimates.

The estimates relied on the assumption that available HCV bio-behavioral data accurately represent PWID within each country. However, data availability and quality varied across countries, and data occasionally came from only a few geographic localities within a country. For instance, in Libya, Ab prevalence was derived from a single high-quality study conducted in the capital city, which reported an exceptionally high Ab prevalence of 94.2%.^73^ This outlier value resulted in a very high incidence rate estimate (Table 1). Conversely, some countries, such as Lebanon, Syria, and Tunisia, reported Ab prevalence levels that were unexpectedly well below the global and MENA average of about 50% (Table 1).^15^^,^^16^

The high variability in Ab prevalence across countries may reflect differences in sampled populations—such as age, injecting duration, geographic location, socio-economic factors, and harm reduction access. Additionally, low Ab prevalence estimates in some countries may result from methodological limitations in the available studies, such as inadvertently including non-injecting drug users due to self-reporting bias or inconsistent protocol implementation. Consequently, these factors could lead to underestimates of HCV incidence among PWID and PWID's contribution to incidence in countries with unexpectedly low Ab prevalence.

The number of PWID in each country plays a critical role in shaping estimates, yet it remains one of the most uncertain model input parameters due to wide variations in estimates.^16^, ^17^, ^18^^,^^21^^,^^22^^,^^26^^,^^27^ These variations arise from differing methodologies and the inherent challenges in estimating the size of this hard-to-reach population.^16^, ^17^, ^18^^,^^21^^,^^22^^,^^26^^,^^27^ We selected what we considered the most representative estimates from those identified in the systematic reviews,^16^, ^17^, ^18^^,^^21^^,^^22^^,^^26^^,^^27^ considering the quality of the methods used, the recency of the data, and alignment with knowledge of the local context of injecting drug use. For example, in Iran, we relied on an estimate from the United Nations Office on Drugs and Crime of 186,686, though other estimates range from 68,500 to 250,000.^16^, ^17^, ^18^^,^^21^^,^^22^^,^^26^^,^^27^ This uncertainty in population size may explain why the estimated HCV incidence among PWID in Iran was higher than that estimated for the total population (Table 1).

Available estimates of HCV incidence in the total population^11^^,^^30^, ^31^, ^32^ were used to compare incidence among PWID to that in the total population. However, these estimates may not reflect the current incidence in countries that have been rapidly expanding DAA treatment programs, such as Egypt,^3^^,^^71^ and they come with their own caveats.^3^^,^^11^^,^^30^, ^31^, ^32^ Due also to expanding treatment programs, the pooled estimate for the viremic rate^29^ may not accurately reflect the current viremic rate in some countries.

In the absence of data, the model assumed that HCV treatment coverage has been negligible among PWID in MENA and that any chronically infected individual can receive treatment. However, treatment may not be indicated for everyone. The interventions were implemented in a manner that is relevant to the MENA context and can inform policy discussions, but this should be considered when interpreting their impact. For instance, reducing needle/syringe sharing and introducing OST were assumed to occur over a very short time frame, whereas treatment was implemented gradually to reach a specific coverage by 2030. This led to varying impacts over time for these interventions (Fig. 1). The study generated numerous estimates, each accompanied by a 95% uncertainty interval, and no multiplicity correction was applied. Consequently, the results should be considered exploratory, and multiplicity should be taken into account when comparing and interpreting differences between estimates, as some differences may appear significant by chance.

This study has several strengths. It fills a gap in estimating HCV rates in MENA, the region most affected by this infection,^1^^,^^2^ and addresses an infection targeted by WHO for elimination by 2030.^3^^,^^10^, ^11^, ^12^ The country-specific parameters were primarily based on systematic reviews and meta-analyses, enhancing the representativeness of the model input. To account for data limitations in the input parameters, multivariable uncertainty analyses were conducted to estimate the uncertainty surrounding the model projections. The study employed an elaborate modeling approach that explicitly accounted for sharing events, sharing groups, and their heterogeneity, as well as a variety of potential interventions. The reliability of the modeling approach was also validated through a sensitivity analysis.

In conclusion, this study highlights the scale of HCV incidence among PWID in MENA, with over 40,000 new infections occurring annually in the 13 countries studied and 10% of PWID being newly infected each year. While the modeled interventions showed potential in reducing incidence, only large-scale or multi-intervention strategies can achieve meaningful reductions in transmission. However, the current response to HCV in MENA remains inadequate to implement such comprehensive interventions, hindered by insufficient political commitment and resource allocation. To meet the WHO's target for HCV elimination by 2030, it is imperative to expand harm reduction programs and increase resource allocation across the region.

## Contributors

MM constructed, co-derived, coded, and parameterized the mathematical model, conducted the modeling analyses, and co-wrote the first draft of the article. GM contributed to the construction, derivation, coding, and parameterization of the model, and also to the writing of the manuscript. LJA conceived the study, designed and co-derived the model, led the modeling analyses, and co-wrote the first draft of the article. MM and LJA accessed and verified all the data. All authors contributed to the discussion and interpretation of the results and to the final draft of the manuscript. All authors have read and approved the final manuscript.

## Data sharing statement

Data generated or analyzed during this study are included in the main text and the supplementary material. The model codes programmed in MATLAB can be obtained by contacting the authors.

## Declaration of interests

The authors declare no conflict of interest.
