# Supplementary material for: Hepatitis C virus transmission among people who inject drugs in the Middle East and North Africa: mathematical modeling analyses of incidence and intervention impact
Source: eClinicalMedicine. 2025 Jan 15;80:103040. doi: 10.1016/j.eclinm.2024.103040 (PMC11786755; doi:10.1016/j.eclinm.2024.103040)
Supplement: Supplementary Figures and Tables [file mmc1.docx]

**SUPPLEMENTARY MATERIAL**

**Table of Contents**

[**Table S1. Model parameters and their values.** 3](#_Toc182483494)

[**Section S1. Mathematical model** 4](#_Toc182483495)

[**Section S2. Heterogeneity in injecting risk behavior** 6](#_Toc182483496)

[**Fig. S1. Gamma distribution of the PWID population into sharing groups of varying sizes, derived from model fitting of HCV antibody prevalence among PWID in each country.** 7](#_Toc182483497)

[**Section S3. HCV treatment with direct acting antivirals** 8](#_Toc182483498)

[**Table S2. Uncertainty intervals of model parameters used in the uncertainty analyses.** 9](#_Toc182483499)

[**Fig. S2. A map of the Middle East and North Africa showing country-specific model-based estimates for A) HCV antibody prevalence, B) HCV incidence rate, and C) the number of new HCV infections among PWID in 2024.** 10](#_Toc182483500)

[**Fig. S3. The 95% uncertainty intervals for the impact of different interventions on HCV prevalence and incidence among PWID in the 13 Middle East and North Africa countries combined, as interventions are introduced on January 1, 2025, with their impact assessed year by year through December 31, 2030.** 13](#_Toc182483501)

[**Fig. S4. Sensitivity analysis for Pakistan. A) Comparison of HCV Ab prevalence prediction from the sensitivity analysis with the Ab prevalence observed in the validation dataset. B-D) Comparison of predictions for key epidemiological outcomes from the sensitivity analysis with those from the main analysis.** 14](#_Toc182483502)

[**References** 15](#_Toc182483503)

#

# **Table S1. Model parameters and their values.**

| **Parameter** | **Symbol** | **Value** | **Source** |
| --- | --- | --- | --- |
| **Biological parameters** |  |  |  |
| HCV Transmission probability per unsterile injection | $\beta$ | 0.035 | 1-5 |
| Effectiveness of prior HCV infection in reducing the risk of HCV reinfection | $g$ | 0.83 | 6,7 |
| **Epidemiological parameters** |  |  |  |
| Total number of PWID | $N$ | Country specific | 8-16 |
| HCV Ab prevalence among PWID | $p_{0}$ | Country specific | 8-15 |
| HCV viremic rate^*^ | $v$ | 0.676 | Pooled estimate from a systematic review^17^ |
| **Behavioral parameters** |  |  |  |
| Annual rate at which PWID stop injecting and become non-PWID | $\sigma$ | 0.1 per year | 9-14 |
| Average number of injections per PWID per year | $n$ | Country specific | 9-14 |
| Average size of sharing group (# of sharing partners) | $m$ | Country specific | Model fitting |
| Proportion of PWID who share injections | $s$ | Country specific | Estimated from the distribution of the sharing group size |
| Proportion of the injections that are shared | $q_{0}$ | Country specific | 9-14 |
| Average number of times a shared needle/syringe is used before disposal | $\delta_{s}$ | Equal to the size of the sharing group, with a maximum value of 10 | 1,9 |
| **Needle/Syringe cleaning parameters** |  |  |  |
| Effectiveness of needle/syringe cleaning in preventing HCV transmission | $\varepsilon_{c}$ | 0.3 | 1,18,19 |
| Proportion of shared injections that are cleaned | $P_{c}$ | 0.15 | 9,12,20,21 |
| **HCV treatment with direct-acting antivirals parameters** |  |  |  |
| HCV treatment effectiveness | $E_{T}$ | 0.9 | 22 |
| Annual rate at which HCV chronically infected persons are treated with direct-acting antivirals | $\pi$ | Country specific and treatment coverage specific | Based on set treatment coverage |

Ab: antibody, HCV: hepatitis C virus, PWID: people who inject drugs.

^*^Proportion of HCV Ab positive persons who are also chronically infected, that is RNA positive.

# **Section S1. Mathematical model**

The cohort mathematical model initially developed by Kwon et al.^1^ was extended and adapted in our earlier study to estimate HIV incidence among people who inject drugs (PWID) in the Middle East and North Africa (MENA).^9^ In this study, the model was further extended to estimate hepatitis C virus (HCV) incidence among PWID in this region. The model assumes that needle/syringe sharing occurs within groups of specific sizes, where PWID share needles/syringes in a random sequence, and each PWID injects once per sharing event. HCV transmission is possible within sharing groups that include at least one chronically infected individual.

The following describes this HCV model:

- Total number of shared injections per year

$$S=Nnsq_{0}$$

- Average number of sharing events per year

$$SE=\frac{Nnsq_{0}}{m}$$

- Probability of having $r$ ever infected persons, $r_{1}$ chronically infected persons among the $r$ infected persons, and $r_{2}=r-r_{1}$ recovered persons, in a sharing group of size $m$

$$P_{r_{1}/r/m}=\left(_{r_{1},r-r_{1},m-r}^{m}\right)\left(vp_{0}\right)^{r_{1}}\left(1-vp_{0}\right)^{r-r_{1}}\left(1-p_{0}\right)^{m-r}=\frac{m!}{r_{1}!\left(r-r_{1}\right)\left(m-r\right)!}\left(vp_{0}\right)^{r_{1}}\left(1-vp_{0}\right)^{r-r_{1}}\left(1-p_{0}\right)^{m-r}$$

- Average number of susceptible individuals who will inject *before* the first chronically infected person in a sharing event

$$U_{b}=\frac{m-(r_{1}+gr_{2})}{r_{1}+gr_{2}+1}$$

- Average number of susceptible individuals who will inject *after* a chronically infected person in a sharing event

$$U_{a}=\left(m-r_{1}-gr_{2}-U_{b}\right)=\frac{m(r_{1}+gr_{2})-(r_{1}+gr_{2})^{2}}{r_{1}+gr_{2}+1}$$

- Average number of susceptible individuals who will use the same needle/syringe *after* a chronically infected (infectious) person in a sharing event

$$U_{s}=\frac{U_{a}}{Sy}=\frac{m(r_{1}+gr_{2})-(r_{1}+gr_{2})^{2}}{r_{1}+gr_{2}+1}\frac{\delta_{s}}{m}$$

where $Sy=m/\delta_{s}$ is the average number of needles/syringes used per sharing event

- Probability of HCV transmission per shared injection

$$\beta_{c}=\beta\left(1-p_{c}\right)+\beta p_{c}\left(1-\varepsilon_{c}\right)=\beta\left(1-p_{c}\varepsilon_{c}\right)$$

- Number of HCV transmissions in the sharing group (that is per sharing event)

$$\begin{matrix} T=\sum_{r=1}^{m-1} \sum_{r_{1}=1}^{r} U_{s}\beta_{c}P_{r_{1}/r/m} \\ =\sum_{r=1}^{m-1} \sum_{r_{1}=1}^{r} \frac{m(r_{1}+gr_{2})-(r_{1}+gr_{2})^{2}}{r_{1}+gr_{2}+1}\frac{\delta_{s}}{m}\beta\left(1-p_{c}\varepsilon_{c}\right)\frac{m!}{r_{1}!\left(r-r_{1}\right)\left(m-r\right)!}\left(vp_{0}\right)^{r_{1}}\left(1-vp_{0}\right)^{r-r_{1}}\left(1-p_{0}\right)^{m-r} \\ =\frac{\delta_{s}\beta\left(1-p_{c}\varepsilon_{c}\right)}{m}\sum_{r=1}^{m-1} \frac{m!}{\left(m-r\right)!}\left(1-p_{0}\right)^{m-r}\sum_{r_{1}=1}^{r} \frac{m(r_{1}+gr_{2})-(r_{1}+gr_{2})^{2}}{r_{1}+gr_{2}+1}\frac{1}{r_{1}!\left(r-r_{1}\right)!}\left(vp_{0}\right)^{r_{1}}\left(1-vp_{0}\right)^{r-r_{1}} \end{matrix}$$

- Total number of HCV transmissions per year (HCV incidence) for all PWID

$$I=SE\times T$$

$$I=\frac{Nnsq_{0}\delta_{s}\beta\left(1-p_{c}\varepsilon_{c}\right)}{m^{2}}\sum_{r=1}^{m-1} \frac{m!}{\left(m-r\right)!}\left(1-p_{0}\right)^{m-r}\sum_{r_{1}=1}^{r} \frac{m(r_{1}+gr_{2})-(r_{1}+gr_{2})^{2}}{r_{1}+gr_{2}+1}\frac{1}{r_{1}!\left(r-r_{1}\right)!}\left(vp_{0}\right)^{r_{1}}\left(1-vp_{0}\right)^{r-r_{1}}$$

- Incidence rate per susceptible person

$$\lambda=\frac{I}{N\left(1-p_{0}+\left(1-v\right)\left(1-g\right)p_{0}\right)}$$

# **Section S2. Heterogeneity in injecting risk behavior**

The original Kwon et al. model^1^ assumed a fixed sharing group size ($m$). This aspect of the model was modified to allow for variability in the sharing group size, reflecting the actual variation observed in PWID populations.^9,12,13^ The sharing group size was assumed to follow a gamma distribution:

$$p\left(m\right)=\frac{1}{\Gamma\left(m\right)\theta^{k}}m^{k-1}e^{-\frac{m}{\theta}}$$

Here, $k$ is the shape parameter and $\theta$ is the scale parameter of this distribution.

Informed by injecting risk behavior data,^9-14^ the sharing group size was assumed to vary between 1 and 10. Additionally, the variance of the gamma distribution was assumed to be equal to its mean, implying that $\theta=1$. The value of $k$, which is equal to the mean of the distribution, was estimated by fitting the model to HCV antibody prevalence among PWID in each country. The distribution by country is found in Fig. S1.

# **Fig. S1. Gamma distribution of the PWID population into sharing groups of varying sizes, derived from model fitting of HCV antibody prevalence among PWID in each country.**


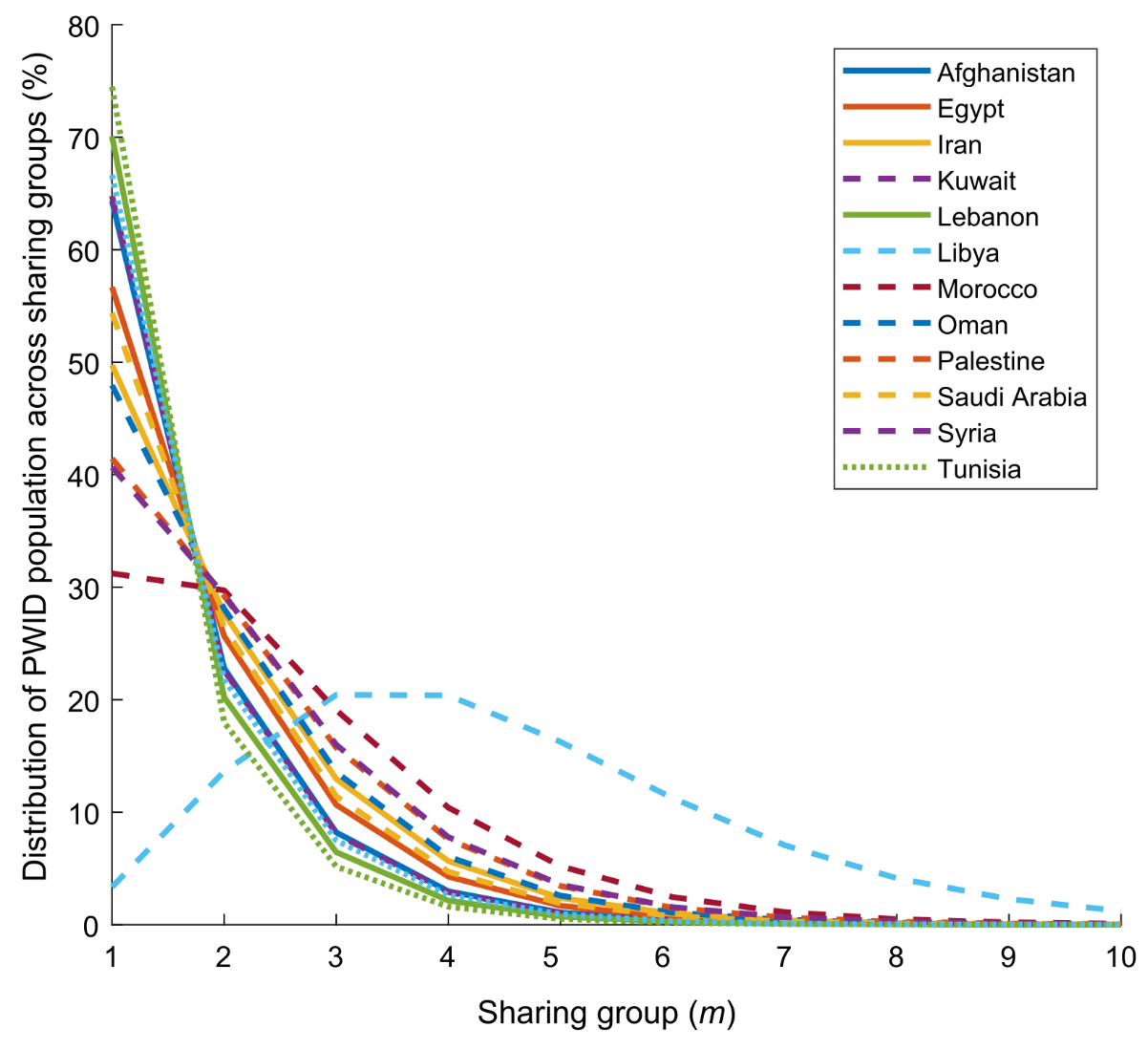


HCV: hepatitis C virus, PWID: people who inject drugs.

# **Section S3. HCV treatment with direct acting antivirals**

Breakthroughs in HCV treatment, namely the development of highly efficacious direct acting antivirals (DAAs), have ushered in a revolution in treating and controlling HCV infection.^23-25^ The model was extended to accommodate treatment with DAAs by administering treatment to chronically infected individuals at an annual treatment rate ($\pi$).

Treatment coverage was defined as the proportion of chronically infected individuals who have been treated, relative to the total number of chronically infected individuals at the year treatment was introduced, in consistency with an existing definition.^22^ The effectiveness of the treatment in real-world conditions ($E_{T}$) was assumed to be 90%.^22^ Successfully treated persons were assumed to be susceptible again to HCV reinfection, but at a reduced risk of reinfection, denoted by $g$.^6,7^

DAA treatment impacts the viremic rate, which represents the proportion of antibody positive individuals who are also chronically infected (RNA positive).^6^ This proportion changes dynamically as PWID are treated in the model simulations. Therefore, the model recalculates the viremic rate at each time step to account for the ongoing treatment effects.

$$v(t)=\frac{Number of chronically infected persons (t)}{Number of ever infected persons (t)}$$

# **Table S2. Uncertainty intervals of model parameters used in the uncertainty analyses.**

| **Parameter** | **Symbol** | **Value** | **Uncertainty interval** | **Source** |
| --- | --- | --- | --- | --- |
| **Biological parameters** |  |  |  |  |
| HCV Transmission probability per unsterile injection | $\beta$ | 0.035 | 0.025-0.050 | 1-5 |
| Effectiveness of prior HCV infection in reducing the risk of HCV reinfection | $g$ | 0.83 | ±30% around the point estimate, with an upper limit of 1.00 | 6,7 |
| **Epidemiological parameters** |  |  |  |  |
| Total number of PWID | $N$ | Country specific | 95% CI of country-specific estimate | 8-16 |
| HCV Ab prevalence among PWID | $p_{0}$ | Country specific | 95% CI of country-specific estimate | 8-15 |
| HCV viremic rate^*^ | $v$ | 0.676 | 0.649-0.703 | Pooled estimate from a systematic review^17^ |
| Number of new HCV infections among the total population per year | - | Country specific | 95% UI of country-specific estimate | 22,26-28 |
| **Behavioral parameters** |  |  |  |  |
| Average number of injections per PWID per year | $n$ | Country specific | ±30% around the point estimate | 9-14 |
| Proportion of the injections that are shared | $q_{0}$ | Country specific | ±30% around the point estimate | 9-14 |
| **Needle/Syringe cleaning parameters** |  |  |  |  |
| Effectiveness of needle/syringe cleaning in preventing HCV transmission | $\varepsilon_{c}$ | 0.3 | ±30% around the point estimate | 1,18,19 |
| Proportion of shared injections that are cleaned | $P_{c}$ | 0.15 | ±30% around the point estimate | 9,12,20,21 |

Ab: antibody, CI: confidence interval, HCV: hepatitis C virus, PWID: people who inject drugs, UI: uncertainty interval.

^*^Proportion of HCV Ab positive persons who are also chronically infected, that is RNA positive.

# **Fig. S2. A map of the Middle East and North Africa showing country-specific model-based estimates for A) HCV antibody prevalence, B) HCV incidence rate, and C) the number of new HCV infections among PWID in 2024.**

**A)**


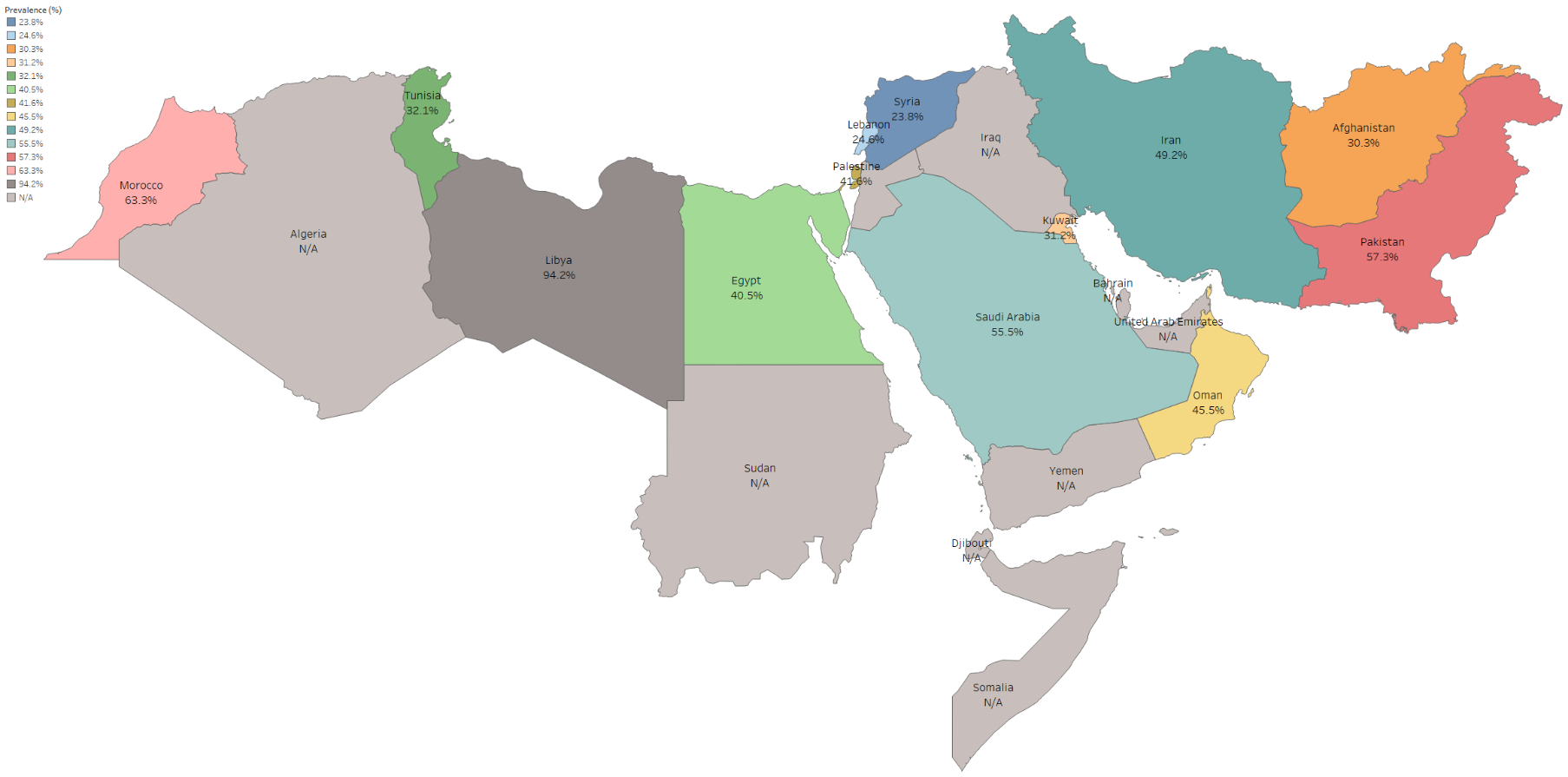


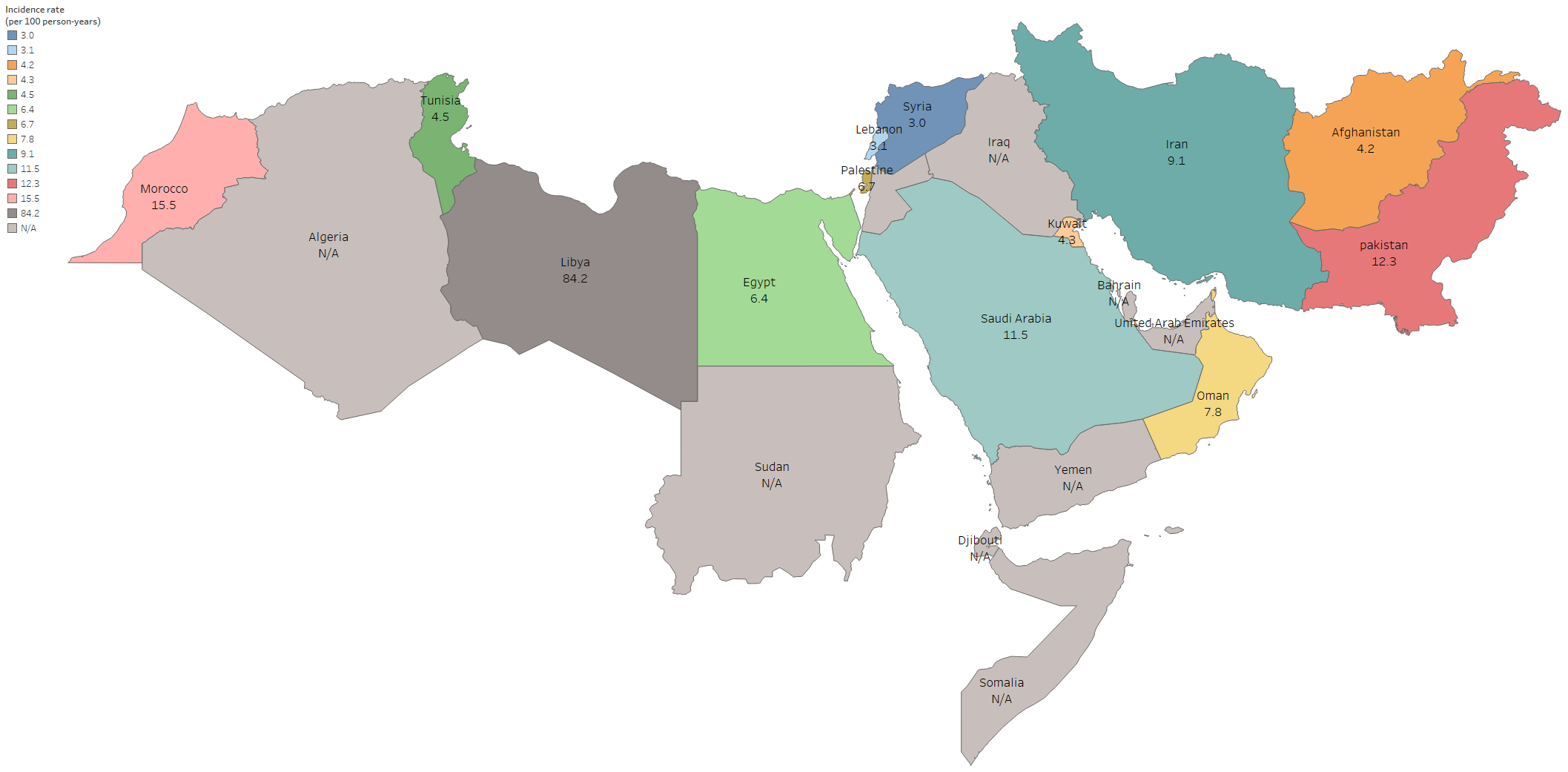


**B)**

**C)**


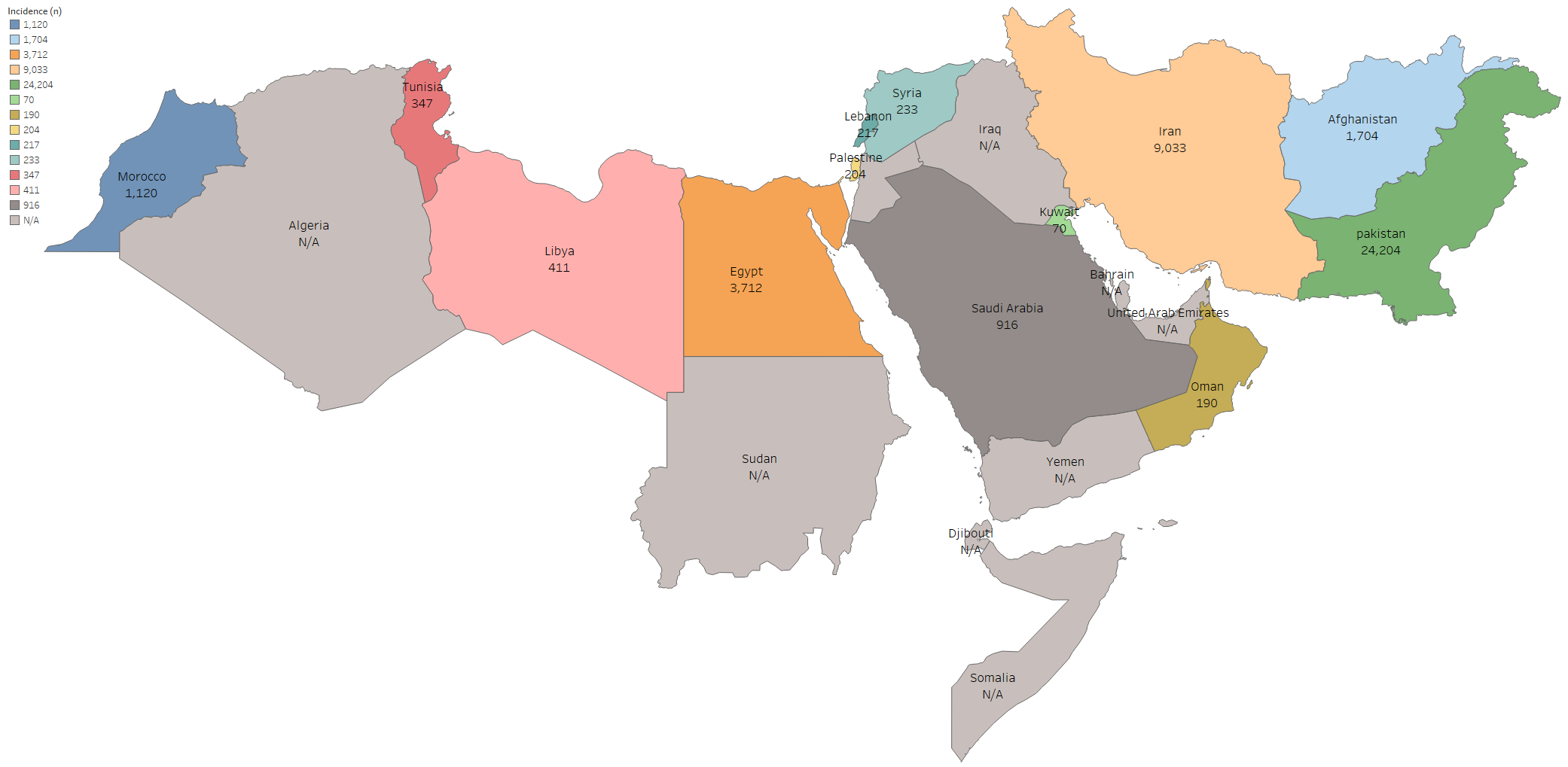


Ab: antibody, HCV: hepatitis C virus, N/A: not applicable as estimate was not possible, PWID: people who inject drugs.

# **Fig. S3. The** **95% uncertainty intervals for the impact of different interventions on HCV prevalence and incidence among PWID in the 13 Middle East and North Africa countries combined, as interventions are introduced on January 1, 2025, with their impact assessed year by year through December 31, 2030.**


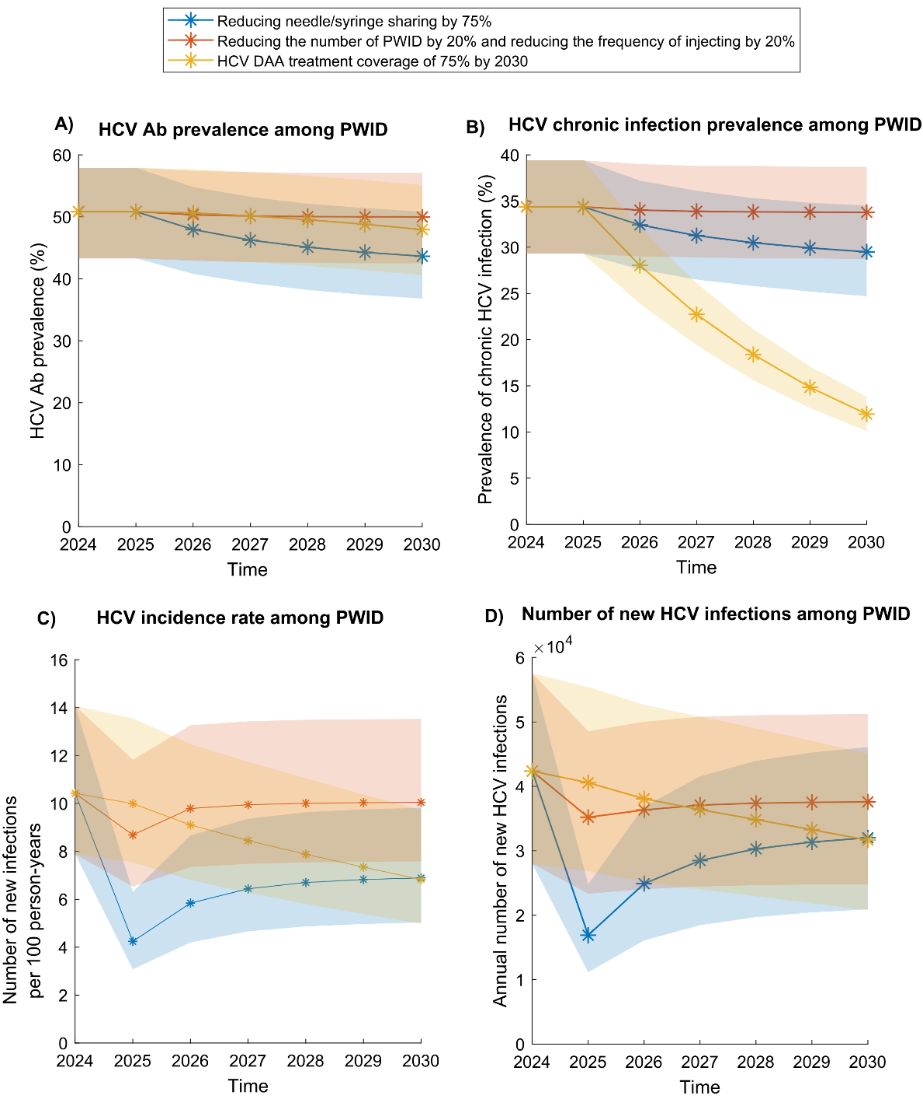


Ab: antibody, HCV: hepatitis C virus, PWID: people who inject drugs.

# **Fig. S4. Sensitivity analysis for Pakistan. A) Comparison of HCV Ab prevalence prediction from the sensitivity analysis with the Ab prevalence observed in the validation dataset. B-D) Comparison of predictions for key epidemiological outcomes from the sensitivity analysis with those from the main analysis.**


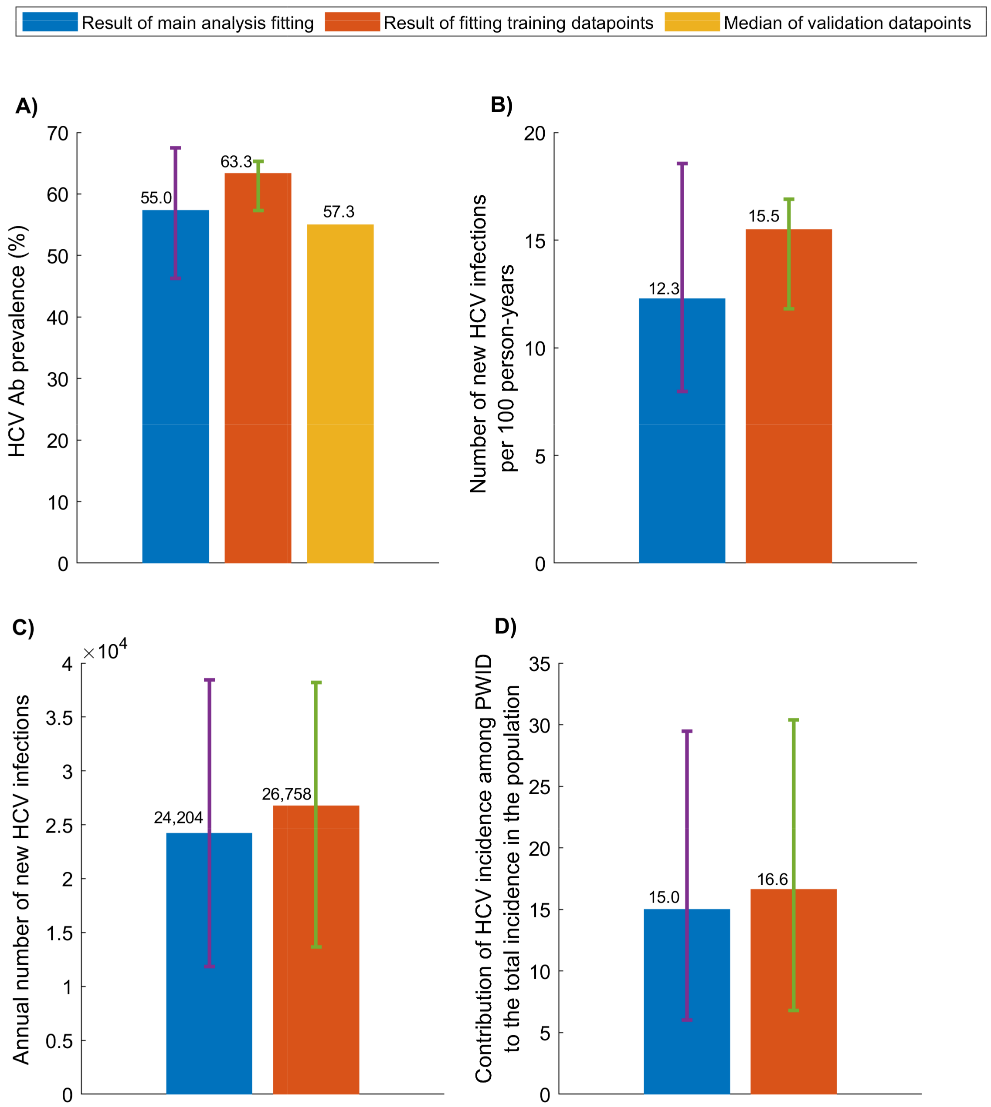


Ab: antibody, HCV: hepatitis C virus, PWID: people who inject drugs.

# **References**

1. Kwon JA, Iversen J, Maher L, Law MG, Wilson DP. The impact of needle and syringe programs on HIV and HCV transmissions in injecting drug users in Australia: a model-based analysis. *J Acquir Immune Defic Syndr* 2009; **51**(4): 462-9.

2. Short LJ, Bell DM. Risk of occupational infection with blood-borne pathogens in operating and delivery room settings. *Am J Infect Control* 1993; **21**(6): 343-50.

3. Kiyosawa K, Sodeyama T, Tanaka E, et al. Hepatitis C in hospital employees with needlestick injuries. *Ann Intern Med* 1991; **115**(5): 367-9.

4. Gerberding JL. Management of occupational exposures to blood-borne viruses. *N Engl J Med* 1995; **332**(7): 444-51.

5. Sodeyama T, Kiyosawa K, Urushihara A, et al. Detection of hepatitis C virus markers and hepatitis C virus genomic-RNA after needlestick accidents. *Arch Intern Med* 1993; **153**(13): 1565-72.

6. Ayoub HH, Chemaitelly H, Omori R, Abu-Raddad LJ. Hepatitis C virus infection spontaneous clearance: Has it been underestimated? *Int J Infect Dis* 2018; **75**: 60-6.

7. Osburn WO, Fisher BE, Dowd KA, et al. Spontaneous control of primary hepatitis C virus infection and immunity against persistent reinfection. *Gastroenterology* 2010; **138**(1): 315-24.

8. Aghaei AM, Gholami J, Sangchooli A, et al. Prevalence of injecting drug use and HIV, hepatitis B, and hepatitis C in people who inject drugs in the Eastern Mediterranean region: a systematic review and meta-analysis. *Lancet Glob Health* 2023; **11**(8): e1225-e37.

9. Mumtaz GR, Awad SF, Feizzadeh A, Weiss HA, Abu-Raddad LJ. HIV incidence among people who inject drugs in the Middle East and North Africa: mathematical modelling analysis. *J Int AIDS Soc* 2018; **21**(3): e25102.

10. Mahmud S, Mumtaz GR, Chemaitelly H, et al. The status of hepatitis C virus infection among people who inject drugs in the Middle East and North Africa. *Addiction* 2020; **115**(7): 1244-62.

11. Mumtaz GR, Chemaitelly H, AlMukdad S, et al. Status of the HIV epidemic in key populations in the Middle East and north Africa: knowns and unknowns. *Lancet HIV* 2022; **9**(7): e506-e16.

12. Mumtaz GR, Weiss HA, Thomas SL, et al. HIV among people who inject drugs in the Middle East and North Africa: systematic review and data synthesis. *PLoS Med* 2014; **11**(6): e1001663.

13. Abu-Raddad LJ, Akala FA, Semini I, Riedner G, Wilson D, Tawil O. Characterizing the HIV/AIDS epidemic in the Middle East and North Africa: Time for Strategic Action. Middle East and North Africa HIV/AIDS Epidemiology Synthesis Project. World Bank/UNAIDS/WHO Publication. Washington DC: The World Bank Press; 2010.

14. Abu-Raddad LJ, Hilmi N, Mumtaz G, et al. Epidemiology of HIV infection in the Middle East and North Africa. *Aids* 2010; **24 Suppl 2**: S5-23.

15. World Health Organization. Epidemiology of hepatitis C virus in the WHO Eastern Mediterranean Region: implications for strategic action. Available from: <https://iris.who.int/handle/10665/336174>. Accessed on: February 12, 2024. 2020.

16. United Nations Office on Drugs and Crime. Drug use in Pakistan in 2013. Available from: <https://www.unodc.org/documents/pakistan/Survey_Report_Final_2013.pdf>. Accessed on: November 20, 2023. 2013.

17. Harfouche M, Chemaitelly H, Kouyoumjian SP, et al. Hepatitis C virus viremic rate in the Middle East and North Africa: Systematic synthesis, meta-analyses, and meta-regressions. *PloS one* 2017; **12**(10): e0187177.

18. Abdala N, Gleghorn AA, Carney JM, Heimer R. Can HIV-1-contaminated syringes be disinfected?: implications for transmission among injection drug users. *JAIDS Journal of Acquired Immune Deficiency Syndromes* 2001; **28**(5): 487-94.

19. Siegel JE, Weinstein MC, Fineberg HV. Bleach programs for preventing AIDS among iv drug users: modeling the impact of HIV prevalence. *American Journal of Public Health* 1991; **81**(10): 1273-9.

20. Corson S, Greenhalgh D, Taylor A, Palmateer N, Goldberg D, Hutchinson S. Modelling the prevalence of HCV amongst people who inject drugs: an investigation into the risks associated with injecting paraphernalia sharing. *Drug Alcohol Depend* 2013; **133**(1): 172-9.

21. Taylor A, Goldberg D, Hutchinson S, et al. Prevalence of hepatitis C virus infection among injecting drug users in Glasgow 1990-1996: are current harm reduction strategies working? *J Infect* 2000; **40**(2): 176-83.

22. Ayoub HH, Mahmud S, Chemaitelly H, Abu-Raddad LJ. Treatment as prevention for hepatitis C virus in the Middle East and North Africa: a modeling study. *Frontiers in public health* 2023; **11**.

23. Flamm SL. Advances in the treatment of hepatitis C virus infection from EASL 2015. *Gastroenterology & Hepatology* 2015; **11**(6 Supplement 3): 1-23.

24. Manns MP, Buti M, Gane E, et al. Hepatitis C virus infection. *Nature reviews Disease primers* 2017; **3**(1): 1-19.

25. Vermehren J, Park JS, Jacobson IM, Zeuzem S. Challenges and perspectives of direct antivirals for the treatment of hepatitis C virus infection. *Journal of hepatology* 2018; **69**(5): 1178-87.

26. Ayoub HH, Abu-Raddad LJ. Impact of treatment on hepatitis C virus transmission and incidence in Egypt: A case for treatment as prevention. *J Viral Hepat* 2017; **24**(6): 486-95.

27. Ayoub HH, Abu-Raddad LJ. Treatment as prevention for hepatitis C virus in Pakistan: mathematical modelling projections. *BMJ Open* 2019; **9**(5): e026600.

28. Ayoub HH, Al Kanaani Z, Abu-Raddad LJ. Characterizing the temporal evolution of the hepatitis C virus epidemic in Pakistan. *J Viral Hepat* 2018; **25**(6): 670-9.
